# Diagnosing point-of-care diagnostics for neglected tropical diseases

**DOI:** 10.1371/journal.pntd.0009405

**Published:** 2021-06-17

**Authors:** Mitasha Bharadwaj, Michel Bengtson, Mirte Golverdingen, Loulotte Waling, Cees Dekker

**Affiliations:** Department of Bionanoscience, Kavli Institute of Nanoscience Delft, Delft University of Technology, Delft, The Netherlands; Foundation for Innovative New Diagnostics (FIND), SWITZERLAND

## Abstract

Inadequate and nonintegrated diagnostics are the Achilles’ heel of global efforts to monitor, control, and eradicate neglected tropical diseases (NTDs). While treatment is often available, NTDs are endemic among marginalized populations, due to the unavailability or inadequacy of diagnostic tests that cause empirical misdiagnoses. The need of the hour is early diagnosis at the point-of-care (PoC) of NTD patients. Here, we review the status quo of PoC diagnostic tests and practices for all of the 24 NTDs identified in the World Health Organization’s (WHO) 2021–2030 roadmap, based on their different diagnostic requirements. We discuss the capabilities and shortcomings of current diagnostic tests, identify diagnostic needs, and formulate prerequisites of relevant PoC tests. Next to technical requirements, we stress the importance of availability and awareness programs for establishing PoC tests that fit endemic resource-limited settings. Better understanding of NTD diagnostics will pave the path for setting realistic goals for healthcare in areas with minimal resources, thereby alleviating the global healthcare burden.

## Author summary

Diagnostic practices are crucial for neglected tropical diseases (NTDs). In this review, we critically discuss each of 24 NTDs as defined in the 2021–2030 roadmap of the World Health Organization (WHO), to identify their specific point-of-care (PoC) diagnostic needs. In doing so, we sketch possible solutions to meet the diagnostic needs by providing realistic technical solutions that will enable early diagnosis of NTDs. We group NTDs in accordance to the common solutions for their PoC diagnostic needs so that joint interventions can be applied for multiple NTDs, in order to lessen the global burden of NTDs.

## Point-of-care diagnostics

The International Organization for Standardization defines point-of-care (PoC) diagnostic testing as “testing that is performed near or at the site of a patient with the result leading to a possible change in the care of the patient” [1]. In practical implementations, a PoC test is a specific and sensitive assessment wherein a user needs to administer a minimum number of steps to obtain an easy-to-interpret, rapid (within a short turnaround time (TAT)), and robust result. PoC tests are used everywhere within the health chain where there is need for a fast diagnostic outcome that is independent from sophisticated, time-consuming, labor-intense, and expensive laboratory procedures. PoC tests are usually designed to function equipment free as portable units with stable reagents that function efficiently within a broad range of environmental settings. Because of these favorable characteristics, PoC diagnostics is also known as “bedside testing,” “remote rapid testing,” “near-patient laboratory testing,” “ancillary testing,” and “decentralized testing” [2–4].

The ease of use of PoC diagnostics clearly exhibits potential to serve as an early diagnostic tool in resource-limited settings. Especially with recent advances in PoC diagnostics based on “lab-on-a-chip” technology, PoC tests can be applied for triage, test of cure, and/or confirmatory diagnostics [5,6]. While a confirmatory test, by definition, verifies a diseased state, a test of cure is used to validate the efficacy of the administered treatment and to distinguish relapse of a disease and reinfections. PoC diagnostics can circumvent issues such as shortage of healthcare staff and underequipped laboratories, thereby improving clinical interventions, which is especially advantageous in resource-limited settings. PoC tests can be field deployable, i.e., they have the ability to be administered in the field within resource-limited settings without the need for a laboratory, which are equipped with sophisticated equipment such as microscopes. Furthermore, PoC diagnostics can also be multiplexed, i.e., simultaneously diagnose more than 1 pathogen from a single sample. Thus, PoC diagnostics, especially when multiplexed, can reduce treatment costs, support disease surveillance, and minimize the unnecessary use of antimicrobials, thereby preventing the emergence of resistant strains [5]. In a nutshell, simple, rapid, reproducible, and robust PoC diagnostic tests are a preferred choice for the diagnosis of neglected tropical diseases (NTDs) that are endemic in resource-limited settings.

**Fig 1:**
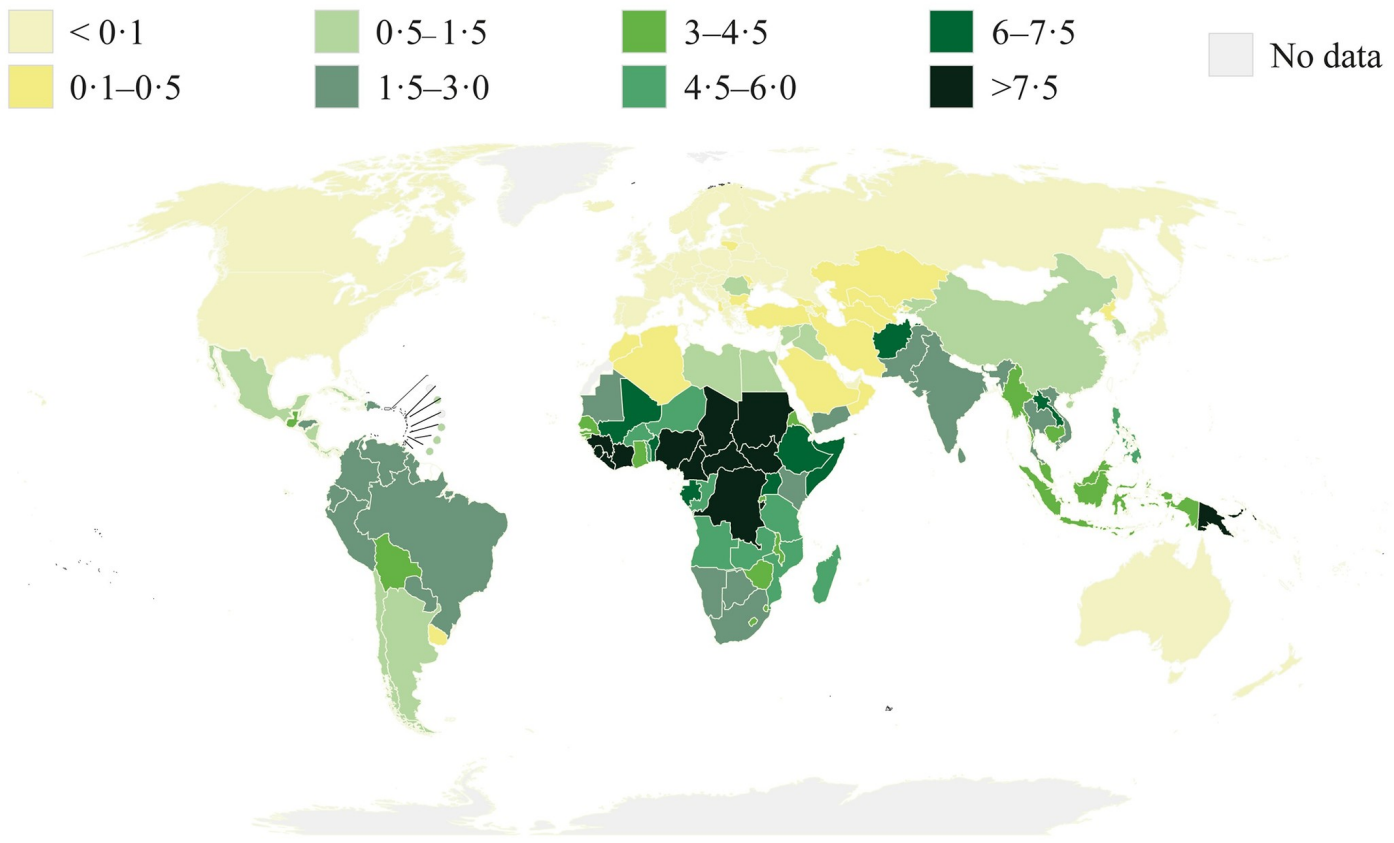
Spread of NTDs. Cumulative DALYs lost of afflicted populations due to the NTDs human African trypanosomiasis, chagas disease, schistosomiasis, leishmaniasis, lymphatic filariasis, onchocerciasis, taeniasis cysticercosis, echinococcosis, dengue, trachoma, rabies, leprosy, and soil-transmitted helminthiases. World map adapted from WHO [8]. DALY, disability-adjusted life year; NTD, neglected tropical disease; WHO, World Health Organization.

NTDs are diseases of poverty that affect more than a billion people worldwide. These communicable diseases are endemic to regions that have limited access to healthcare, and, hence, despite available treatments, they can be fatal. These diseases are referred to as “neglected” as they receive inadequate attention, e.g., in terms of research funding, when compared to other diseases such as malaria, human immunodeficiency virus, and tuberculosis (TB) [7]. NTDs represent a disease burden of at least 22 million disability-adjusted life years (DALYs) lost, roughly half the burden of TB or malaria [7]. The World Health Organization (WHO) has identified 24 NTDs, for control and elimination in the 2021–2030 roadmap [8]. Although tremendous progress has been made in combatting NTDs over the past decade, they still prevail in high incidence numbers, significantly contributing to numerous DALYs of afflicted populations (Fig 1). In effect, NTDs pose the biggest disease burden to developing tropical economies [9–13].

WHO’s Sexually Transmitted Diseases Diagnostics Initiative (SDI) developed the ASSURED criteria as a benchmark for diagnosis, which stands for affordable, sensitive, specific, user-friendly, rapid and robust, equipment-free, and deliverable to end users [14]. Recent advances in the PoC diagnostics highlighted the issues with data and sample collection; therefore, new term “REASSURED” was coined comprising of real-time connectivity and ease of specimen collection and environmental friendliness to existing ASSURED [15]. Furthermore, disease-specific guidelines called “target product profiles” have been developed for certain NTDs. Unfortunately, despite multiple guidelines such as these for NTDs, there is a lack of commercially viable diagnostic tests, i.e., certified by regulatory commissions for medical use. Thus, while scientific research has been conducted for recognizing new targets and novel approaches for NTDs diagnoses, the tests based on such research are typically not developed further for their commercial use [16–20]. The need of the hour is commercially available, field-deployable PoC tests to facilitate early disease diagnosis, which is crucial for NTDs control and treatment, and for designing strategies for the gradual elimination of these endemic diseases.

In this review, we first discuss different technical approaches for PoC diagnostic tests, i.e., their basic working principle, advantages, and limitations. Next, in the core of this review, we outline the status quo of PoC NTD diagnostics, highlighting the implementation need for various PoC NTD diagnostics. We end with a discussion and recommendations for future developments to further accelerate the goal of achieving efficient NTD diagnostics.

## Three different approaches for PoC diagnostics

**Fig 2:**
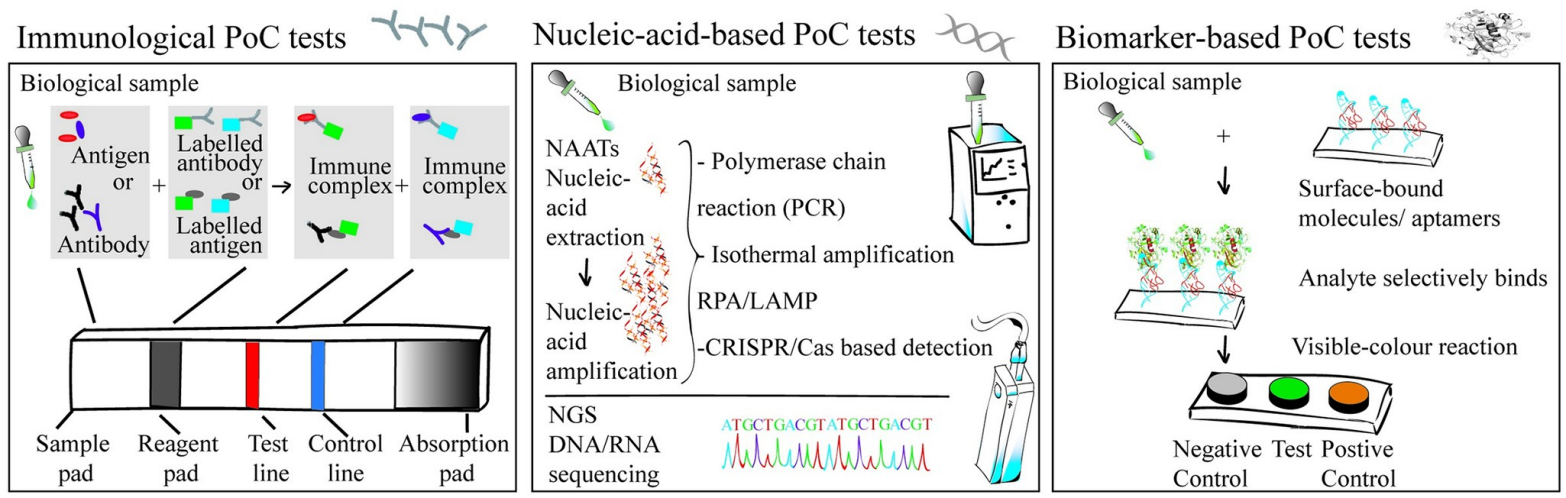
Three types of PoC diagnostic tests. A biological sample (such as blood, urine, saliva, sweat, etc.) can be utilized for various types of PoC diagnosis. **Left**: immunological PoC test. A biological sample is dropped unto the sample pad of a lateral flow assay, which acts as a filtering unit to sieve out unnecessary constituents. Upon administration of a reaction buffer (or its automated release), the analyte flows through the reagent pad, wherein an antigen–antibody complex is formed. Driven by capillary action, this complex migrates to the next zone with control and test lines. While the appearance of a visible color at the test line confirms the infection, the control line signal ensures the test functionality. **Middle**: nucleic acid–based PoC test. Here, genetic material of a pathogen serves as the analyte. DNA/RNA from the pathogen is extracted from infected host cells or circulating cell free within the clinical sample (body fluids). While extracted RNA is first reverse transcribed to obtain cDNA, extracted DNA can be directly amplified using PCR or using isothermal amplification (e.g., RPA or LAMP), typically in a fully automated portable unit. In some systems, the amplified DNA is then used for CRISPR/Cas recognition or other downstream processing to yield a diagnostic result within a lateral flow assay or a microfluidic lab-on-a-chip device. NGS can also be utilized to identify specific diseases using a portable sequencer. **Right**: biomarker-based PoC test (other than antigen biomarkers). A biological sample is administered onto the test pad that in this case has specific surface-bound molecules such as aptamers that target the analyte. Upon successful interactions, a visible color readout is obtained. The test can be in the form of separate wells. LAMP, loop-mediated isothermal amplification; NGS, next-generation sequencing; PCR, polymerase chain reaction; PoC, point-of-care; RPA, recombinase polymerase amplification.

At the heart, PoC diagnostics is an approach to identify and possibly quantify a specific analyte. Based on the variety of biomolecular ways for detection of specific analytes, PoC tests can be classified into immunological tests, nucleic acid–based tests, and other biomarker-based tests (Fig 2)—which we describe below.

### Immunological PoC tests

Immunological PoC tests detect the presence of an antigen (or its molecular counterpart) or an antibody that is generated as an immune response to an infection. In these PoC tests, an antibody/antigen immobilized on a solid substrate binds to the specific analyte, forming an immune complex that subsequently generates a signal that is visible to a naked eye (Fig 2). The specific antigen–antibody interactions that are at the core of these immunosensors allow for both qualitative detection and quantitative assessment.

Immunological PoC tests have numerous advantages and hence are among the most commonly used tests. In addition to a quick TAT of approximately 15 minutes, these PoC tests do not require sample preparation, and, thus, a clinical sample can be administered onto the test pad directly. Immunological PoC tests can be field deployable as they are simple, rapid, easy to use, equipment free, and robust, thus allowing administering at decentralized locations in an NTD-endemic region. Moreover, immunological PoC tests are cost effective compared to the other PoC approaches [21] discussed below.

Immunological PoC tests also have limitations [22]. Due to cross-reactivity, autoantibodies and rheumatoid factors can reduce the efficacy of antigen-based immunological PoC tests, as these molecules can specifically bind to free antigens in the sample and thereby block them from interacting with the specific antibody immobilized on the immunosensor. Antigen quantity also critically influences early disease diagnosis. While low antigen levels might result in false negatives, high levels of the antigen can cause “prozoning” wherein excess antigen occupies most of the antibody-binding sites and interferes with the downstream steps, thus resulting in false negative test results (hook effect). Antigen denaturation is another major factor affecting the lifetime and functionality of antigen-based PoC tests. Furthermore, antibody-based tests in general cannot be used as a confirmatory test or as a test of cure because they can provide false positive results due to the presence of antibodies that persist postinfection in the body. Furthermore, the growth cycle of a pathogen can influence the immune response. While fast-growing pathogens could elicit an immune response within a few days of infection, slow-growing pathogens could take weeks before a detectable amount of antibody can appear in the bodily fluid, resulting in false negative results. In many cases, pathogens can evade the immune system and reside in the body for weeks to months before a detectable immune response is generated (asymptomatic carriers). Likewise, the overall efficiency of an immunological PoC test may be influenced by the varying immune responses of individuals within a population, especially when afflicted populations are malnourished or have compromised immune systems due to comorbidities. Finally, multiplexing immunological PoC tests is challenging as the test can favor one analyte over another, affecting the analytical efficiency. Immunological PoC tests are also not ideal to diagnose species specificity and to prescribe optimal treatment choices, for example, in antibiotic therapy. Therefore, immunological PoC tests are mostly used as screening tests to select patients for subsequent confirmatory diagnosis [2,23].

### Nucleic acid–based PoC tests (molecular PoC tests)

Nucleic acid–based PoC tests probe for the presence of genetic material of the pathogen (DNA or RNA) to diagnose specific diseases. While specific amplification of the pathogens genetic material for disease diagnoses can be performed using nucleic acid amplification tests (NAATs), mapping of the pathogen sequence for diagnosis is commonly done by next-generation sequencing (NGS) of the DNA/RNA (Fig 2). Commercially available PoC NAATs are based on the polymerase chain reaction (PCR) that occurs in a closed automated device that utilizes prepackaged single-use integrated cartridges filled with reagents for the nucleic acid amplification [2]. The user simply needs to load the sample fluid and start the reaction by pressing a button. The entire PCR reaction then proceeds inside the device, and the result is displayed within a TAT of 20 to 60 minutes on a screen. Such small, portable, and battery/power-operated PCR units can be used for the detection of a range of pathogens by simply adapting the cartridges [24,25].

With the increasing demand for field-deployable PoC molecular diagnostic tests, new isothermal NAATs are being designed that, unlike PCR, do not require a thermocycler but instead function at a constant temperature, for example, at 37°C or even at room temperature [26,27]. Examples of commonly used isothermal NAATs are strand displacement amplification, loop-mediated isothermal amplification (LAMP), and recombinase polymerase amplification (RPA), techniques that allow the amplification and detection within a TAT of 20 minutes. As these methods may suffer from relatively low specificity, novel Clustered Regularly Interspaced Short Palindromic Repeats (CRISPR)-based systems are being developed to function downstream of isothermal NAATs to enhance the specificity [17]. Capable of multiplexing, such detection systems could potentially become the best PoC molecular diagnostic toolbox. Owing to advancements in in-silico approaches, unique DNA targets can be identified for every pathogen. Thus, nucleic acid–based PoC tests can be multiplexed and have the potential to detect many diseases simultaneously (*n* ≥100 with microarrays) [28]. However, so far, they have been restricted to laboratory diagnostics, and further development is needed to adapt them into field-deployable PoC tests [29–33]. Portable NGS devices are one of the most promising candidates for efficient PoC diagnostics in the near future. Similar to NAATs, innovative Next Generation Sequencing (NGS) technologies have been developed for nontrained personnel with simplified handling, fast and accurate results, and reduced sample volumes [34].

Nucleic acid–based PoC tests provide a range of advantages. Given their very high sensitivity and specificity, they can detect even a single pathogen in a biological sample. Furthermore, they lend themselves well for multiplexing. These PoC tests can serve as confirmatory diagnostics as they can identify the infection etiology, microbial resistance, and the extent of an infection (virulence)—thereby accelerating the choice of treatment [35]. Limitations of nucleic acid–based PoC tests are mostly found in the pretreatment of the samples. Owing to the extreme sensitivity of nucleic acid–based tests, uncontaminated input samples need to be administered in a closed automated unit to avoid false positive results [36]. A further challenge can be to ensure accurate detection of traces of microbial nucleic acids within the pool of other components in the biological sample, say, the human genome, to avoid false negative results. A related challenge is to ensure specificity by identifying a unique target for the pathogen’s DNA that does not exhibit any homology with other genomes in the sample. At this point in time, nucleic acid–based PoC tests are too costly for resource-limited settings [37]. On top of that, additional costs due to hardware infrastructure for data handling may be a concern in such settings [34].

### Biomarker-based PoC tests

Biomarkers are biological molecules (other than antigens) that occur naturally in living beings and can be used to indicate a diseased state when deviations occur in their concentrations from the physiologically normal value. In this review, we refer to biomarkers as any molecule other than the antigen itself or the antibody that is elicited in response to the infection (cf. Section 2.1) or the pathogen’s nucleic acid material (cf. Section 2.2). While a plethora of biomarkers has been identified for infectious disease diagnosis [38,39], C-reactive protein is so far the only biomarker that is available as a commercial PoC test for infectious diseases, and it is used for selecting effective antimicrobial treatment in acute respiratory tract infections [2].

New opportunities for biomarker-based PoC tests are emerging with aptamers—specific oligonucleotide sequences that bind a biomarker with high affinity. Since aptamers are synthetically produced, unlike antibodies, they are highly reproducible, cost effective, and easy to modify. Aptamers are thermostable and can be reversibly denatured, making them a potent biosensor for PoC tests (Fig 2). These “aptasors” have, for example, been developed for aptamer-linked immunosorbent assays or enzyme-linked oligonucleotide assays also known as enzyme-linked aptamer assays [40–42].

The main advantages of biomarker-based PoC tests are that they can provide fast and accurate results [38,42]. Quite some research is ongoing to develop biomarker-based PoC tests for confirmatory diagnosis. A major disadvantage is the higher costs in the development and commercial applicability of biomarker-based PoC tests compared to immunological PoC tests [2].

## PoC tests for neglected tropical diseases

Owing to the advantages of PoC tests, they are highly preferred for diagnosing NTDs in resource-limited settings. NTD diagnostics is challenging since these diseases often strongly relate to the context, which includes the needs and capabilities of the end users, available resources and infrastructure, extent of disease endemicity, and treatment options [20]. Successful implementation of NTD PoC diagnostics must therefore consider, in addition to the technical efficiency, the context-specific requirements.

In this review, we comprehensively summarize the available literature for each of the 24 NTDs that were identified in the 2021–2030 roadmap of WHO [8], focusing on their current diagnostics, specifically on PoC tests if available, and the implementation need for future PoC diagnostics. An extensive overview is provided in the Supporting information. A detailed evaluation of the status quo of PoC diagnostics for all 24 NTDs facilitated us to formulate prerequisites of a PoC test that would fit the disease-specific context. Next to recognizing the required technical improvements, complimentary needs were identified based on the treatment and follow-up routines for the NTDs. A summary of the resulting information on the current PoC tests (both commercially available tests as well as tests in the pilot phase) and implementation needs for future PoC tests is presented in Table 1.

**Table 1:** NTD-specific status quo of existing PoC test and future implementation needs for PoC tests.

Disease name	Current PoC tests	Implementation needs	References
Foodborne trematodiases	✔ 2 antigen-based tests and 1 nucleic acid–based test for *Fasciola gigantica* ✔ 3 nucleic acid–based tests for *Opisthorchis viverrini* ✔ 1 antigen-based test for *Paragonimus* spp. ✔ 1 antigen-based and 1 nucleic acid–based test for *Clonorchis sinensis*	✔ Effective field-deployable sample preparation as DNA extraction is limited to a laboratory ✔ Quality assessment and field validation for all the current PoC tests	[52–54]
Taeniasis cysticercosis	None reported	✔ Quality assessment and field validation of the antigen-based proof-of-principle test (see Supporting information) ✔ Novel biomarker identification ✔ Reliable field-deployable PoC test	[55,56]
Echinococcosis	✔ Several commercial antibody tests for cystic and alveolar echinococcosis (e.g., VIRAPID HYDRATIDOSIS, ADAMUCE, RIDASCREEN *Echinococcus* IgG test) ✔ 1 nucleic acid–based LAMP test for cystic echinococcosis	✔ More reliable PoC tests as current tests have low sensitivity and cannot detect inactive cysts ✔ Confirmatory diagnostic tests for humans (test of cure) ✔ Field-deployable screening tests for dogs	[52,57]
Rabies	✔ Several commercial immunological tests (e.g., Vet-o-test Rabies Ag and Antigen Rapid Rabies Ag test kit)	✔ Novel circulating biomarker identification for dogs and humans to mitigate invasive sampling (brain tissue) ✔ Field-deployable PoC test for humans ✔ Quality assessment and field validation for all current PoC tests	[58–60]
Chromoblastomycosis	None reported	✔ Field-deployable nucleic acid–based test as species specificity will aid effective treatment	[61]
Leishmaniasis (cutaneous)	✔ CL Detect Rapid Test (antibody-based test) ✔ Loopamp Leishmania detection kit (nucleic acid–based) ✔ palmPCR (handheld battery-operated device) (nucleic acid–based)	✔ Field-deployable sample preparation is required for the Loopamp Leishmania Detection Kit and palmPCR ✔ Quality assessment and field validation for all current PoC tests	[62–65]
Mycetoma	None reported	✔ Field-deployable nucleic acid–based test as species specificity will aid effective treatment (bacterial treatment is more effective than fungal treatment)	[66]
Human African trypanosomiases (*rhodesiense*)	None reported	✔ Novel circulating biomarker identification ✔ Field-deployable nucleic acid–based species-specific tests will aid effective treatment	[67–69]
Buruli ulcer	✔ Pilot immunological tests and molecular tests (LAMP and RPA)	✔ Quality assessment and field validation of the pilot tests ✔ Field-deployable sample preparation ✔ Confirmatory test (test of cure) is critical in cases of coinfections	[70–72]
Schistosomiasis	✔ Schisto POC-CCA for *Schistosoma japonicum* and *Schistosoma mansoni Schistosoma* ICT IgG-IgM rapid test ✔ UCP-LF CAA assay	✔ Field-deployable nucleic acid–based test to enable the detection of low parasitaemia ✔ Field-deployable antigen-based test to detect *Schistosoma haematobium*. ✔ Ultrasensitive field-deployable antigen-based test to detect low intensity *Schistosoma*	[73–76]
Chagas disease	✔ Several commercial immunological tests (e.g., Chagas STAT-PAK assay, Chagas Detect Rapid Plus test (antigen-based), Trypanasoma Detect	✔ Field-deployable nucleic acid–based test to detect congenital chagas disease	[13,77,78]
Leishmaniasis (visceral)	✔ IT-LEISH Kit ✔ Kalazar Detect ✔ *Onsite Leishmania AB* Rapid Test ✔ VL-LFD device	✔ A robust test for use in East Africa, where the IT-LEISH Kit is not effective ✔ Quality assessment and field validation of the VL-LFD device ✔ Field-deployable nucleic acid–based test (test of cure), which is crucial for the diagnosis of post-kala-azar (PKDL)	[79–81]
Lymphatic filariasis	✔ Alere Filariasis Test Strip (antibody-based) ✔ SD BIOLINE Lymphatic Filariasis IgG4 (antigen-based) ✔ Brugia Rapid (antigen-based)	✔ Field-deployable PoC test without cross-reactivity with *Loa loa* ✔ Multiplexed test with *Loa loa* ✔ Novel circulating biomarker identification	[82,83]
Chikungunya	✔ SD BIOLINE Chikungunya (antigen-based) ✔ Chikungunya IgM Combo Rapid Test CE (antigen-based)	✔ Quality assessment and field validation ✔ Field-deployable PoC test without cross-reactivity with dengue ✔ Multiplexed test with dengue ✔ Novel circulating biomarker identification	[84,85]
Scabies	✔ Burrow ink test ✔ Handheld dermatoscopy test	✔ A standardized diagnostic procedure will prevent misdiagnoses and delayed treatment	[86–88]
Onchocerciasis	✔ SD BIOLINE *Onchocerciasis* IgG4 (antigen-based) ✔ SD BIOLOINE *Onchocerciasis*/LF biplex test with *Onchocerca volvulus* and *Wuchereria bancrofti* (antigen-based)	✔ Field-deployable PoC test without cross-reactivity with *Loa loa* as this coinfection affects the treatment regimen ✔ Novel circulating biomarker identification ✔ Confirmatory test (test of cure) to aid surveillance in the current eradication era	[89]
Human African trypanosomiases (*gambiense*)	✔ SD BIOLINE HAT and SD BIOLINE HAT 2.0 (antigen-based) ✔ HAT Sero K-Set (antigen-based)	✔ Quality assessment and field validation ✔ Field-deployable nucleic acid–based species-specific tests will aid effective treatment ✔ Confirmatory test (test of cure)	[90–92]
Snake bite envenoming	✔ 1 test for 5 Australian species (antibody-based)	✔ PoC test that is multiplexed for species that are geographically distinct ✔ A reliable PoC coagulation analyzer to diagnose coagulopathy	[13,93–95]
Dengue	✔ Multiple commercial antigen and antibody-based tests (e.g., SD BIOLINE Dengue Duo Rapid Test Kit and ASSURE Dengue IgA Rapid test (antigen-based)	✔ Field-deployable PoC test without cross-reactivity with the Zika virus ✔ Novel circulating biomarker identification ✔ Multiplexed test with the Zika virus	[13,96–98]
Dracunculiasis	None reported	✔ Field-deployable nucleic acid–based test for humans, for canines, and for copepods (crustaceans) to test bodies of water for surveillance purposes during the current eradication era	[8,99]
Leprosy	None reported	✔ Quality assessment and field validation of the biomarker-based proof-of-principle test (see Supporting information)	[39]
Soil-transmitted helminthiases	✔ Kankanet (smart microscopy tool)	✔ Improved algorithms for other infectious species as Kankanet can only detect *Ascaris lumbricoides* ✔ Field-deployable test to detect resistance to treatment and test of cure ✔ Multiplexed tests for other helminth infections	[8,100]
Trachoma	✔ 1 lateral flow test (antigen-based) ✔ Grading tool	✔ Quality assessment and field validation ✔ PoC test with enhanced sensitivity to detect acute infections ✔ Confirmatory test (test of cure)	[101–103]
Yaws	✔ DPP	✔ Quality assessment and field-deployable nucleic acid–based test to distinguish between yaws and syphilis ✔ Multiplexed test with syphilis	[104–107]

For each NTD, an overview is given of current PoC tests and the most urgent implementation needs. Current PoC tests are either listed according to their commercial product names or as tests in the pilot phase. NTDs that have several commercial PoC tests, such as echinococcosis, have been described in detail in the Supporting information.

DPP, Dual Path Platform; LAMP, loop-mediated isothermal amplification; NTD, neglected tropical disease; PKDL, post-kala-azar dermal leishmaniasis; PoC, point-of-care; POC-CCA, point-of-care circulating cathodic antigen; RPA, recombinase polymerase amplification; UCP-LF CAA, up-converting phosphor lateral flow circulating anodic antigen; VL-LFD, visceral leishmaniasis–lateral flow device.

We found that 6 NTDs do not have any commercially available PoC tests, 16 NTDs are currently diagnosed with immunological PoC tests, and 7 NTDs are currently diagnosed with nucleic acid–based tests. The 6 NTDs for which no commercially available PoC diagnostic test is available are taeniasis, *rhodesiense* human African trypanosomiasis, dracunculiasis, and the skin NTDs leprosy, mycetoma, and chromoblastomycosis. For taeniasis, a new analyte for a PoC test must be identified that can serve as a biomarker for the presence of tapeworms and their larval forms, not only in humans but also in pork meat and water, to prevent ingestion of contaminated food and water. Likewise, for dracunculiasis, testing of water bodies for disease surveillance necessitates a field-deployable nucleic acid–based test. For chromoblastomycosis and mycetoma, novel PoC tests, ideally nucleic acid-based, are required that can recognize causal species for efficient treatment. Although immunological-based PoC tests are available for the diagnosis of rabies, schistosomiasis, chagas disease, lymphatic filariasis, chikungunya, snake bite envenoming, dengue, trachoma, and yaws, these are found to be suboptimal (see Supporting information), and further development of more reliable tests for the diagnosis of these NTDs is needed. The most common need among these NTDs is found to be the field deployability, followed by confirmatory diagnosis and test of cure. Notably, field deployability includes requirements such as reliability (stable storage at room temperature), reproducibility (batch-to-batch variation), costs, and accessibility to the test. Both immunological-based and nucleic acid–based PoC tests are available for foodborne trematodiases, echinococcosis, buruli ulcer, leishmaniasis (both visceral and cutaneous), onchocerciasis, and *gambiense* human African trypanosomiasis. Interestingly, despite the availability of a PoC test, these tests need to be further optimized for use in the field, preferably for applications as simple as “under a tree.” Dedicated field studies (quality assessment and field validation) must be conducted to evaluate the performance of such PoC tests.

**Fig 3:**
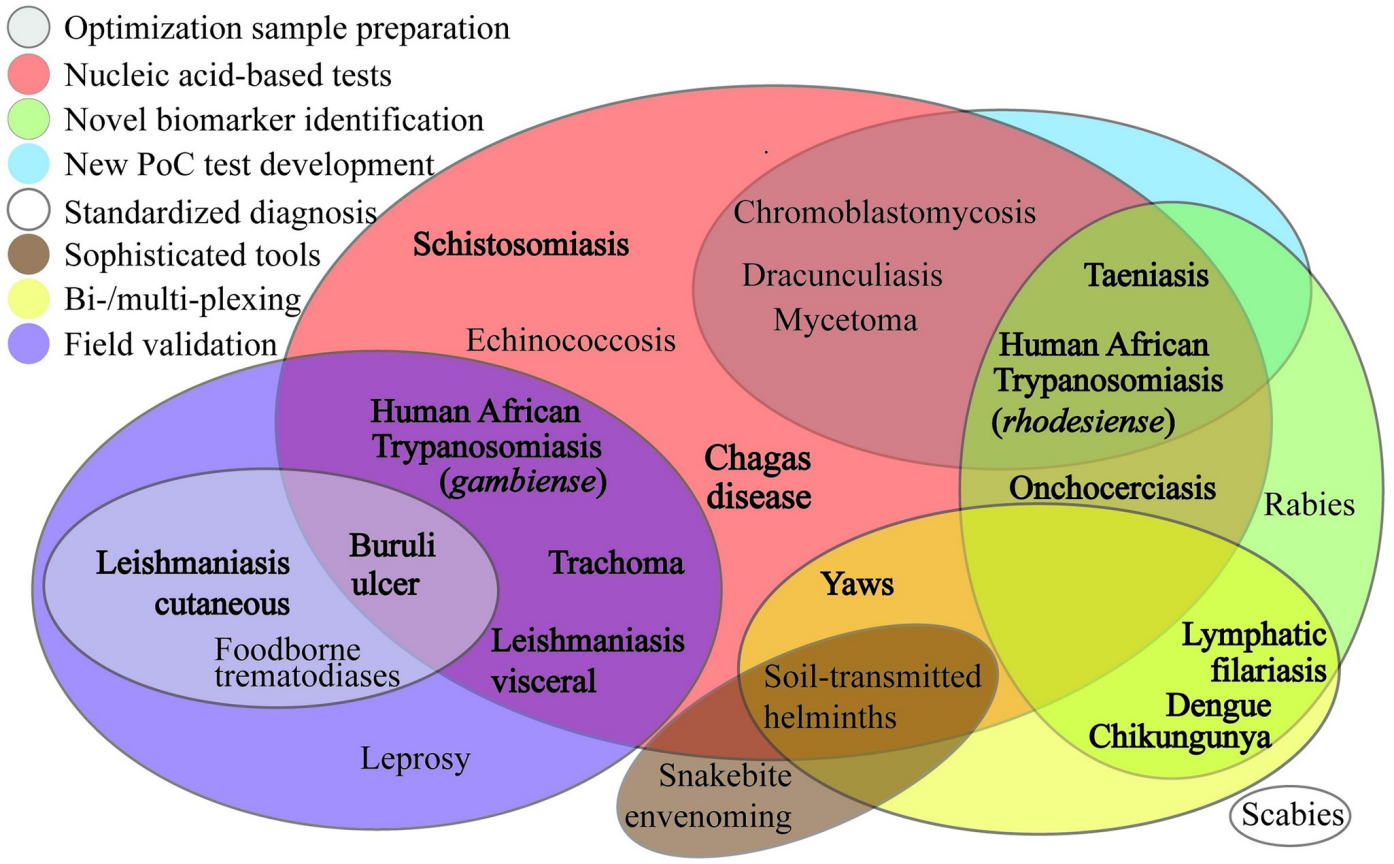
Venn diagram depicting the PoC implementation needs for various NTDs. After reviewing the 24 NTDs (see Supporting information and Table 1), NTDs were placed in the colored circles based on their PoC implementation need. The legends indicate the particular diagnostic need of the NTD. NTDs in bold depict priority for PoC diagnoses as set in WHO’s 2021–2030 roadmap [8]. NTD, neglected tropical disease; PoC, point-of-care; WHO, World Health Organization.

We grouped these 24 NTDs based on our observations on common technical requirements for developing novel PoC tests for efficient diagnosis (see Fig 3). The demand for confirmatory diagnosis and a test of cure was one of the most common implementation needs as can be deduced from the requirement for robust PoC tests utilizing nucleic acid–based detection (red ellipse) or recognizing novel biomarkers (green ellipse). Along with field validation of existing PoC tests (purple), robust confirmatory species-specific tests are required for efficient and effective diagnosis of visceral leishmaniasis and *gambiense* human African trypanosomiasis. Likewise, new sensitive and specific tests such as nucleic acid–based/biomarker PoC tests are required for the diagnosis of schistosomiasis particularly in case of low parasitemia. Nucleic acid–based PoC tests would also be the ideal solution for diagnosing congenital and asymptomatic chagas disease. For taeniasis and *rhodesiense* human African trypanosomiasis, PoC tests with novel biomarker detection are required since there are currently no PoC tests available for these NTDs. For lymphatic filariasis, dengue, and chikungunya, existing PoC tests are inefficient as they are cross-reactive with other infectious diseases (Table 1). Febrile illnesses dengue and chikungunya are often misdiagnosed as they occur as a seasonal outbreak within Zika-endemic regions, and, hence, novel PoC tests should be capable of multiplexing to distinguish between these viral illnesses and thereby accelerate the diagnoses and minimize treatment delays. Similarly, onchocerciasis diagnosis would benefit from a confirmatory test and a test of cure. For the skin NTDs cutaneous leishmaniasis and buruli ulcer, sample preparation is a major concern as skin samples must be obtained in a sterile environment. Thus, novel PoC tests that could function with (noninvasive) surface scrapings of the skin tissue are required for efficient diagnosis here.

**Fig 4:**
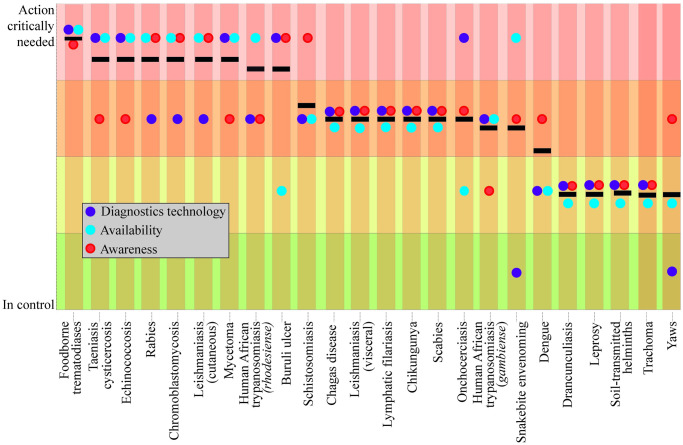
Diagnostic insufficiencies for NTDs. NTDs were ranked in the order of diagnostic insufficiencies (black bars) as deduced from 3 parameters: diagnostics technology (purple), availability (blue), and awareness (red), and scored for “action critically needed” to “in control.” Information was obtained from a literature survey, including comprehensive recent reports from WHO, particularly WHO 2021–2030 roadmap [8] and the diagnostic technical advisory group for NTDs report [20]. Data were critically analyzed to gather diagnostic insufficiencies for individual diseases. NTD, neglected tropical disease; WHO, World Health Organization.

Analysis of the status quo of NTDs diagnostics practices highlighted that, although technically adequate PoC tests might exist for certain NTDs, it is the disease unawareness and test unavailability in the endemic regions that often delimit the effective use of PoC tests. Henceforth, based on WHO’s 2021–2030 roadmap [8] and diagnostic technical advisory group for NTDs [20], and our comprehensive literature survey (Supporting information), we further analyzed the NTDs for disease awareness, tests availability, and diagnostic technology insufficiencies (Fig 4). To rank the diagnostic insufficiencies of the 24 NTDs, we identified 3 parameters: awareness, availability, and diagnostic technology, and scored the needs for each NTD from “in control” to “action critically needed.” For many of the NTDs, we found that the major diagnostic insufficiency was contributed by test unavailability and lack of disease awareness, as seen for the 8 NTDs in the far left of Fig 4. For example, diagnosis of snake bite envenoming and rabies is delayed due to preference to traditional healing practices because there is lack of awareness and existing tests are not readily available. Similarly, for skin NTDs including chromoblastomycosis, mycetoma, and cutaneous leishmaniasis, misdiagnosis due to lack of awareness results in delayed diagnosis and treatment. Likewise, diagnostic insufficiencies for foodborne trematodiases, echinococcosis, scabies, and other ectoparasites could be addressed by ensuring disease awareness and existing test availability, and novel PoC diagnostics should be developed only if efficient diagnosis is still not achieved. Summing up, it is pivotal to urgently address the issues of disease awareness and test availability because they act as the main obstacles in the introduction of novel PoC tests in endemic settings.

In addition to disease unawareness and tests unavailability, other limitations and barriers can hinder successful implementation of PoC tests for NTDs [17,43]. PoC tests can potentially yield inaccurate test results due to poor analytical quality or improper sample handling—indicating a need for staff training for proper test protocols, result documentation, and device maintenance. Furthermore, the clinical relevance of a PoC test over conventional reference diagnostic standards has been a persistent concern among medical practitioners. Since “first-generation” lateral flow rapid immunochromatographic tests for infectious diseases were often questioned for their analytical quality over laboratory counterparts, new sophisticated PoC tests are often subjected to some mistrust by medical practitioners in remote regions [44]. Surprisingly, rapid on-the-spot self-testing or testing by health workers who cannot provide the adequate post test counseling can in practice also have unexpected effects. For certain diseases, a positive result of a PoC test can significantly affect the mental and social well-being of the patient. NTDs with a high psychosocial burden includes human African trypanosomiasis, chagas disease, skin NTDs (leprosy and cutaneous leishmaniasis), or diseases that may cause lifelong physical disabilities such as an amputation of affected body part in case of buruli ulcer or mycetoma. Therefore, in addition to providing a PoC test, an effective diagnostic protocol for adequate awareness and counseling is needed among the health workers in the local communities to provide psychosocial support. Moreover, since diagnosis of NTDs is currently often based on physical examination and suspicion due to disease endemicity in the region, a negative PoC test result can challenge and jeopardize the relationship between the patient and the healthcare worker or the medical practitioner [45], possibly even yielding more general mistrust toward western medicine and a shift of healthcare-seeking behavior toward traditional healers.

Finally, there are issues of cost and political context. The implementation of PoC tests for NTDs should follow standardized clinical pathways to ensure the judicious use of the PoC test [1]. Lack of technical support and maintenance is often an issue, especially in remote settings. Since sophisticated research is needed for the development of PoC diagnostic tests, many tests are far from cost effective, especially for NTDs wherein all interventions are donor dependent and generally administered free of charge. A lack of incentive and low return on investments are common reasons for neglect relating to NTDs diagnoses in comparison to other infectious diseases. Costs due to supply chain, transport, and storage also hinder the sustainable use of a PoC test for NTDs [46]. Moreover, clinical trials (validation) of PoC diagnoses for NTDs are difficult to conduct for various reasons, including persistent migration and poor healthcare-seeking behaviors among marginalized populations. Besides, adoption of new technologies in endemic regions often faces long delays in obtaining regulatory approval and in subsequent implementation in healthcare systems. [47] Health services in endemic regions are often insufficiently equipped for the management of NTDs and a lack of governmental support to implement innovative PoC tests for NTDs can be challenging, especially when new PoC tests do not confer a direct economic advantage over existing laboratory-based diagnostic test in the region [48]. Summing, implementation of PoC diagnostics for NTDs presents significant challenges.

## Discussion and conclusions

From our review of the status quo of PoC diagnostic tests and practices for all of the 24 NTDs mentioned in WHO’s 2021–2030 roadmap, we determined the diagnostic needs and formulated prerequisites of the relevant PoC tests. We identified that confirmatory diagnosis and a test of cure are the most common implementation needs for all NTDs. A paradigm shift is needed toward developing fully automated robust PoC tests for confirmatory NTDs diagnostics and possibly test of cure. Additionally, due to a vicious circle of disease endemicity, i.e., poor healthcare-seeking behavior due to extreme poverty, lack of awareness, persistent migration, and poor livelihood of marginalized populations, NTD diagnostic tests must be brought to the doorsteps of remote communities. Field-deployable PoC tests at the local community level for NTD screening would benefit treatment and aid in reaching the desired goal of NTD elimination. Multiplexed PoC diagnostics for related illnesses would not only ensure correct diagnosis, identifying comorbidities and benefitting timely treatment, but also eliminate problems due to misdiagnoses and reduce financial burdens upon seeking healthcare. For multiplexing PoC devices, the disease combination could result from various considerations. For example, multiplexed tests can be made for diseases that are geographically overlapping, especially when treatments differ in case of comorbidities, or for diseases that present overlapping clinical presentations such as febrile illnesses. However, the research and development involved in such multiplexed tests are challenging as unique and non-cross-interfering targets must be identified for every pathogen, and, therefore, the cost of a multiplexed test can be larger. Yet, in the bigger picture, multiplexing is a cost-beneficial practice in the long run, as diagnosing multiple infections using a single sample reduces costs incurred due to multiple independent tests and corresponding treatment delays.

Developing technically advanced PoC diagnostic tests does not automatically ensure that they will be routinely used in endemic regions. Therefore, in addition to technically improving PoC diagnostic tests, it is vital that future interventions focus on creating awareness programs and developing appropriate logistics to ensure that the necessary PoC tests are available within endemic regions for those NTDs where the major diagnostic need lies in a lack of awareness and test unavailability. In other words, it is important to determine if an NTD requires a novel PoC test or if efforts should enhance awareness or increase availability of existing PoC tests. Research should thus only focus on developing novel PoC tests for those NTDs where the technical inefficiencies of existing diagnostic tests present the main barrier, but local awareness of the NTD must be enhanced with progressive knowledge transfer and capacity building initiatives to engage and empower the local communities. Lessons learned from successful diagnostic strategies should be customized and applied to NTDs. For example, the success stories from malaria control programs can be adapted to related febrile illnesses such as dengue and chikungunya.

Since multiple NTDs are often prevalent in endemic regions, common solutions can be combined and integrated. In almost every individual NTD study, we observed that complex multiparametric factors are present that involve challenges and potential strategies to combat them. For example, skin NTDs such as buruli ulcer, cutaneous leishmaniasis, leprosy, mycetoma, yaws, and onchocerciasis all require common diagnostics practices and case management strategies [49] and thus can be integrated for disease awareness, disease mapping, and training of the healthcare workers. Likewise, linking logistics to ensure availability of the diagnostics tests in remote regions presents opportunities for integration, i.e., the supply chain for multiple tests can be combined so that the tests can be delivered together from national hospitals to the peripheral health facilities in the endemic regions.

In general, a comprehensive approach with a 360° view of the NTDs (and other diseases) spread within a region, and their context is needed to devise a reliable, sustainable, robust solution for combating NTDs. NTD-afflicted communities have inadequate basic amenities such as water, food, housing, etc., which cause further spread of diseases in endemic regions. For example, water often plays a critical and common role in the spread of NTDs. Schistosomiasis is a waterborne disease, wherein parasites multiply in water snails, and, hence, water acts as a reservoir of this disease vector. Likewise, trachoma is a water-scarce disease, wherein person-to-person transmission is due to a lack of water for basic hygiene. Thus, for combating schistosomiasis and trachoma, a common solution could be to ensure the availability of quality water along with basic sanitation and hygiene practices (WASH) [50]. Similarly, insect-transmitted diseases such as dengue, chikungunya, trypanosomiasis, and leishmaniasis can be combatted together by integrating vector control programs like insecticide-treated nets, indoor insecticide spraying, treatment of water reservoirs, and awareness programs. Hence, multiple NTDs that have direct correlation with the basic needs such as clean water, sanitation, nutrition, vector management activities, veterinary public health activities, etc. can be combated together by providing common solutions. In essence, instead of vertical interventions that target one disease at a time, multiple NTDs can be tackled simultaneously by horizontally integrating related interventions to achieve common objectives and thereby aid the prevention and control of NTDs.

**Fig 5:**
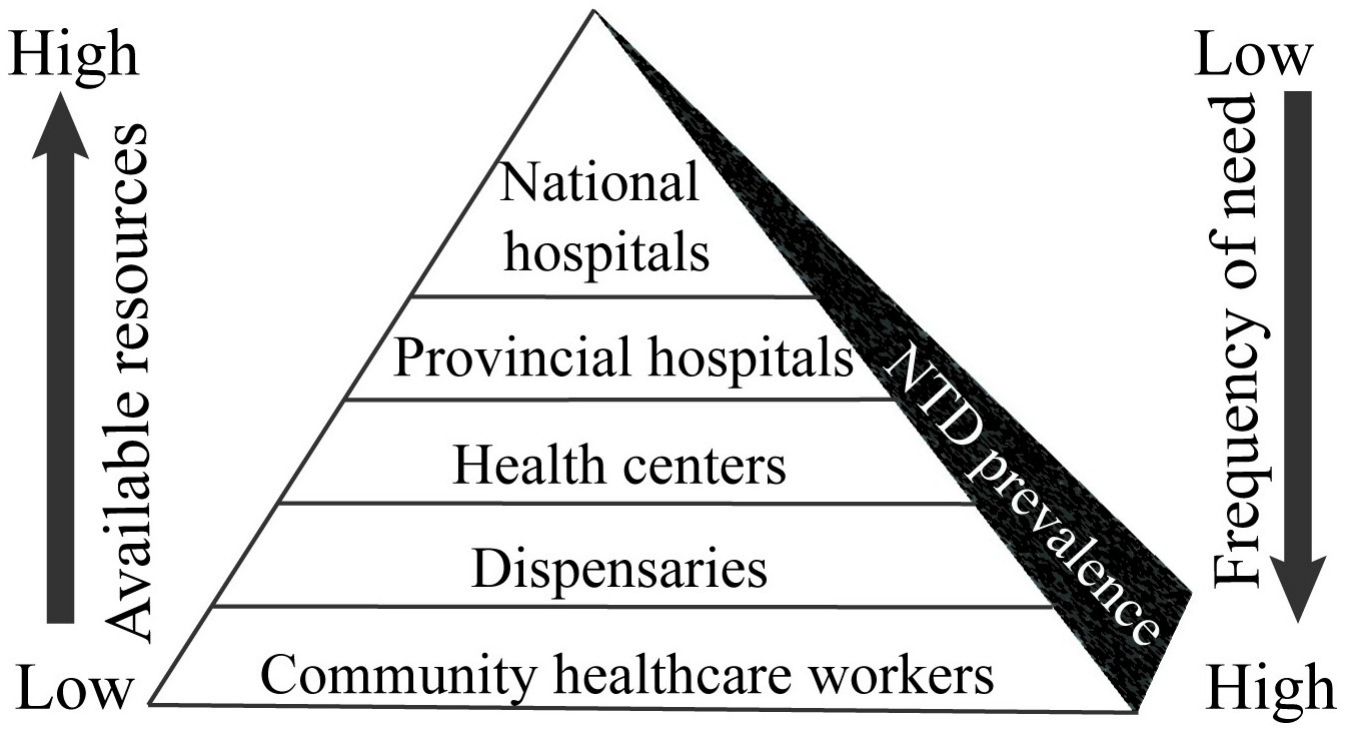
The healthcare paradox for NTDs in endemic regions. At the lowest level, the healthcare system in the NTD endemic region has minimally trained CHWs capable of providing referrals to a level up such as local dispensaries with limited resources including technicians and/or midwives, followed by health centers with trained medical professionals, laboratory space, equipment, and/or inpatient wards. These CHWs at the base of the pyramid also play a vital role in facilitating mobile health outreach services, thereby bridging the gap between patients and medical facilities. One level further up is adequately equipped provisional hospitals followed by fully functional national hospitals at the top of the pyramid. Notably, the burden of NTDs is highest at the lowest healthcare level. This indicates the healthcare paradox, i.e., the highest NTD needs occur at the base of the pyramid where resources are inadequate, and, thus, the disease spreads further due to unmet healthcare needs. To interrupt this vicious cycle of disease endemicity, easy-to-use NTD diagnostics should be introduced to the CHWs at the lowest level. Bringing NTD diagnostics at the doorstep of the endemic populations will ensure efficient disease diagnosis and treatment, reduce costs, and thereby alleviate the healthcare burden. CHW, community healthcare worker; NTD, neglected tropical disease.

Finally, for the successful control and desired eradication of an NTD, customized PoC diagnostic solutions need to be devised. The healthcare paradox in NTD-endemic regions is that the highest need for healthcare occurs in areas with minimum resources, i.e., at the lowest level of healthcare system, thus posing significant challenges for patients that seek adequate healthcare (Fig 5). Thus, diagnostic solutions should not only be patient centric and abide to context-specific requirements as highlighted above but they should also be in line with subsequent treatment approaches and healthcare system in general. For example, if treatment requires hospitalization, a robust PoC diagnostic test for screening of the disease would suffice in the field, and follow-up tests can be performed at the hospital. However, for NTDs wherein treatment can be limited to medication that can be administered at home or at local minimally equipped healthcare center, a robust confirmatory PoC diagnostic test would be needed. In a nutshell, a context-specific holistic approach is necessitated that combines early disease diagnosis, effective treatment interventions, and control strategies for multiple diseases at the same time [12,45,46,48,49].

Summing up, to accelerate the global efforts for management of NTDs, crosscutting strategies spanning across all NTDs interventions from the development of new PoC diagnostic tests to treatment and control, to improvements such as WASH, nutrition, and living standards, to issues of cost and political context need to work hand in hand. Especially now that emerging diseases cause global pandemics, consolidated initiatives such as the “one health” approach [51] is fitting for the timely management of NTDs to alleviate the global healthcare burden.

## Methods

A literature review was conducted using an exploratory search strategy of electronic databases, including Google Scholar, PubMed, Medline, Google Patents, Google Books, Web of Science, Espacenet, Pubget, Scopus, IEEE Xplore, Open Content, WHO websites, CDC website, and Wikipedia. For the entire review, we searched for research articles with the keywords relevant to each section of the review. We used Medical Subject Headings (MeSH) terms for disease names and causal species names, in combination with keywords including “diagnostics,” “rapid diagnostic tests,” “commercial tests,” “point-of-care tests/testing,” “NTD diagnostics,” “bedside testing,” “remote rapid testing,” “near-patient laboratory testing,” “ancillary testing,” and “decentralized testing” in different combinations. We analyzed all articles and reports published and included those relevant to the scope of this review. Additional articles were obtained by citation tracking of review and original articles. For the comprehensive overview provided in the Supporting information, NTDs were equally divided among authors, (i.e., 4 to 5 NTDs per author) to produce an overview of the PoC diagnosis for each NTD (see Supporting information), yielding a brief description of the disease, the current diagnostics, and PoC tests, if available. Thereafter, the overview of PoC diagnosis of the NTDs was reviewed by the first authors, and implementation needs were deduced. Implementation needs refer to specific technical requirements that would support the translation from proof-of-principle tests to field-deployable PoC tests. Any discrepancies were discussed among all authors until a consensus was reached.

## Supporting information

S1 Text Supporting information file. This file contains an overview of all 24 NTDs identified by WHO 2021–2030 roadmap (based on diagnostic requirements and is an extension of the well-known list of 20 NTDs). These NTDs are presented in the order of their diagnostic insufficiencies (Fig 4) wherein for each NTD, we provide a brief 1-page overview with a brief introduction with a description of the disease, current diagnostics, and PoC test, if available. Implementation need. In the description, the NTDs appear in the same order as for the figures and the table in the main manuscript. NTD, neglected tropical disease; PoC, point-of-care; WHO, World Health Organization.
(DOCX)
