# Supplementary material for: Diagnosing point-of-care diagnostics for neglected tropical diseases
Source: PLoS Negl Trop Dis. 2021 Jun 17;15(6):e0009405. doi: 10.1371/journal.pntd.0009405 (PMC8211285; doi:10.1371/journal.pntd.0009405)
Supplement: S1 Text — This file contains an overview of all 24 NTDs identified by WHO 2021–2030 roadmap (based on diagnostic requirements and is an extension of the well-known list of 20 NTDs). These NTDs are presented in the order of their diagnostic insufficiencies (Fig 4) wherein for each NTD, we provide a brief 1-page overview with a brief introduction with a description of the disease, current diagnostics, and PoC test, if available. Implementation need. In the description, the NTDs appear in the same order as for the figures and the table in the main manuscript. NTD, neglected tropical disease; PoC, point-of-care; WHO, World Health Organization. (DOCX) [file pntd.0009405.s001.docx]

**SUPPLEMENTARY INFORMATION**

connected to manuscript:

**Diagnosing point-of-care diagnostics for neglected tropical diseases**

Mitasha Bharadwaj*^,^ Michel Bengtson*^,1^, Mirte Golverdingen, Loulotte Waling, and Cees Dekker^$^

^1^Department of Bionanoscience, Kavli Institute of Nanoscience Delft, Delft University of Technology, Delft, The Netherlands.

* Equal contribution

^$^ Corresponding author: c.dekker@tudelft.nl

**Content of this supplementary information file**

This file contains an overview of all 24 Neglected Tropical Diseases (NTDs) identified by WHO 2021-2030 roadmap (based on diagnostic requirements, and is an extension of the well-known list of 20 NTDs). These NTDs are presented in the order of their diagnostic insufficiencies (Figure 4) wherein for each NTD, we provide a brief one-page overview with

- A brief introduction with a description of the disease,
- Current diagnostics,
- Point-of-care (PoC) test, if available.
- Implementation need.

In the description below, the NTDs appear in the same order as for the figures and the table in the main manuscript.

**Foodborne trematodiases**

Foodborne trematodiases are caused by infections with trematode worms. Infectious species are *Clonorchis sinensis*, *Opisthorchis viverrini*, *Opisthorchis felineus*, *Fasciola hepatica*, *Fasciola gigantica*, and *Paragonimus species.* [1] Foodborne trematodiases are transmitted through raw and undercooked food (fish, aquatic vegetables, crabs and crayfish) that are infected with trematode larvae. Infection with trematode worms causes severe abdominal pain, malaise, inflammation and fibrosis of the liver, possibly fatal bile duct cancer (clonorchiasis and opisthorchiasis), colic pain and jaundice from fascioliasis (liver fluke infection), chronic cough and chest pain, shortness of breath (dyspnea), and fever from paragonimiasis (lung fluke infection). Foodborne trematodiases are found throughout the world, however, African and South‑East Asian countries have the highest burden. Approximately 200,000 people are infected annually, causing ~7,000 deaths and ~2 million DALYs (2016).[2] Children under the age of five account for approximately one-third of deaths. Foodborne trematodiases are treated by mass drug administration (MDA) of anthelminthic medication, such as praziquantel and triclabendazole, in endemic areas.[3] These infections are of great economic and public health importance, especially veterinary significance as livestock are affected as well.[4]

Current diagnostics

Diagnostics for foodborne trematodiases is done through imaging (X-ray, magnetic resonance imaging (MRI)), serology (ELISA), histopathology (definitive individual diagnosis) and parasitological microscopy techniques,[1] such as the detection of eggs or the parasite itself in stool samples using the Kato-Katz thick smears method.[4,5] Faecal egg detection and worm recovery are the reference standard diagnostic method. Polymerase chain reaction (PCR) tests are still at the experimental stage and not routinely used, especially in resource-limited settings.[4] Although microscopy is mainly used for diagnoses, it has low sensitivity and requires an experienced user.

PoC test

A few PoC tests have been developed for foodborne trematodiases[4] There are two antigen-based^[[1]](#footnote-1)^ tests and one nucleic acid-based test for fascioliasis, three nucleic acid-based tests for opisthorchis, one antibody test for paragonimiasis, and one antibody and one nucleic acid-based test for clonorchiasis.[4] However, many of these PoC tests have not been validated in the field, and are thus not routinely used. Furthermore, the DNA extraction step for these tests is limited to laboratories. There is an immunochromatography test (ICT) kit for *Clonorchis sinensis* and *Opisthorchis viverrini* that detects IgG antibodies, and does not require any equipment, but it still needs to be validated in the field before it can be routinely used.[6]

Implementation need

These PoC tests need to be validated in field in order for them to be routinely used in endemic regions. Current field deployable tests fail due to ineffective sample preparation (*i.e.,* not cracking the eggs to release the DNA) which leads to false negative test results.[1] There is an urgent need for sample preparation methods that do not rely on the use of a laboratory.[4]

**Taeniasis/cysticercosis**

Taeniasis is an intestinal infectious disease that is caused by adult tapeworms from the genus *Taenia*. Cysticercosis is a particular taeniasis infection that is caused by *Taenia solium* (pork tapeworm). Among all the tapeworm infections, *T. solium* infections cause the most significant health issues. Infection with *T. solium* occurs when raw infected pork is eaten, via the fecal-oral route or via ingesting contaminated water. Most infected patients are asymptotic or presented with a mild headache. Although the larval cystic stage is relatively harmless in most tissues, the cysts can cause neurocysticercosis (NCC) when they develop in the nervous system. The brain and eye tissues are particularly susceptible to the formation of these cysts.[7] The most frequent symptoms of NCC are epilepsy and seizures, intracranial hypertension, hydrocephalus, vascular damage and stroke, cognitive deficit and depression.[8] Cysticercosis is endemic in low income countries with poor sanitation and domestic pig raising in Africa, Asia, and Latin America. Approximately 5.5 million people are infected worldwide, causing ~28,000 deaths, and ~2.8 million DALYs. The total number of people suffering from NCC is estimated to be between 2.56 – 8.30 million worldwide.[9] Taeniasis is treated by MDA of anthelminthic medication, such as niclosamide or praziquantel as a single dose, or albendazole for 3 days.[9]

Current diagnostics

Diagnosis of NCC is based on neuroimaging supported by immunological diagnosis.[7,8] Neuroimaging via computer tomography (CT) and magnetic resonance imaging (MRI) show the presence, number, location, and size of the *T.solium* tapeworms in the brain. MRI provides better images of small lesions and CT scans show the calcification in the brain that is caused by the parasite, indicating that preferably both MRI and CT tests are used. However only CT scans are used in poor endemic countries.[8] Patients with a viable cyst infection test positive, while patients that only have calcified lesions test negative when using immunological diagnostic tests. However, cross reactions with antibodies of other parasites requires the use of immunological diagnostic tests in combination with neuroimaging.[8,10] Molecular techniques based on PCR to detect *T. solium* specific genes have also been developed, although they are not routinely used in endemic regions.

PoC test

There are currently no commercial PoC diagnostic tests available for taeniasis.[11] There is a proof‑of‑principle immunological antigen-based lateral flow assay (*i.e.,* that detects antibodies in a sample) with high sensitivity and specificity. However, the antigens that are used in this test are difficult to produce.[12]

Implementation need

Novel biomarker identification and a newly developed PoC test (which could be biomarker or nucleic‑acid‑based) are urgently needed to contribute to the effective diagnosis of NCC in endemic regions.

**Echinococcosis**

Echinococcosis is a zoonotic parasitic disease that occurs in humans in two main clinical forms, cystic echinococcosis (CE) and alveolar echinococcosis (AE),[13] which are caused by the tapeworms *Echinococcus granulosus and Echinococcus multilocularis*, respectively.[14] Adult worms develop in the intestines of dogs, foxes, and other carnivores. The eggs are then ingested by humans *via* contaminated food, and larvae develop in organs such as the liver, lungs, kidneys, spleen, bones and muscle tissue. Both forms of this disease first develop asymptomatically as incubation periods can last for many years, until the larvae develop and trigger clinical symptoms, which can be fatal when left untreated. Chronic symptoms include cough, chest pain, and shortness of breath. Symptoms depend on the location of the cysts. Non-symptoms include anorexia and weakness.[14] Echinococcosis is distributed in every continent except Antarctica and more than 1 million people are infected worldwide. The highest prevalence occurs in rural areas where cattle are slaughtered. The 2015 WHO Foodborne Disease Burden Epidemiology Reference Group (FERG) estimated there are approximately 19,300 deaths and approximately 871,000 DALYs reported globally each year.[2] Echinococcosis is of both public health and economic importance, as infected cattle have decreased milk production, reduced fertility, and decrease in hide value. Echinococcosis is expensive and complicated to treat, as surgery and chemotherapy are often required. The cysts can be removed with the PAIR surgical technique (Puncture, Aspiration, Injection, Re-aspiration).[15]

Current diagnostics

Ultrasonography imaging is the reference standard for the diagnosis of both clinical forms of echinococcosis in humans. This is complemented with MRI and CT scans. Histopathology (definitive individual diagnosis) and molecular assays (PCR) can also be used. Serological tests are also able to detect antigens to support the imaging diagnosis.[15]

PoC test

There are several commercial immunological PoC tests (antigen-based) for both clinical forms of this disease,[16]examples include VIRAPID HYDRATIDOSIS, ADAMU-CE and RIDASCREEN Echinococcus IgG test, and one LAMP (Loop-mediated isothermal amplification) detection test (NADH 1-LAMP) has also been developed for CE.[4] The current PoC tests are used only to complement imaging diagnosis as they have poor sensitivity in detecting inactive cysts.

Implementation need

More reliable PoC tests are needed that can serve as test-of-cure for humans (antigen or nucleic-acid-based). Field‑deployable PoC tests for dogs would also contribute to the control of this disease.[16]

**Rabies**

Rabies is a one of the oldest known viral infections that is caused by the rabies virus (RABV) and by other lyssaviruses. The bite of rabid animals (especially dogs) and the saliva of the infected host are mainly responsible for the transmission of rabies. Wildlife like raccoons, skunks, bats, and foxes are main reservoirs for rabies. The incubation period is highly variable from 2 weeks to 6 years (average 2–3 months). Even though rabies causes severe neurologic signs and can be fatal, neuropathological lesions are relatively mild. Rabies can cause progressive and possibly fatal neurological impairment. It affects all warm-blooded animals and the disease is present throughout the world, and endemic in many countries except on islands such as Australia and Antarctica. More than 60,000 people die every year due to rabies, and approximately 15 million people receive rabies post-exposure prophylaxis (PEP) annually. The WHO reports ~1.6 million DALYs due to rabies. Vaccination with live attenuated or inactivated viruses, DNA and recombinant vaccines can be done in endemic areas. Treatment includes a fast-acting rabies injection of immune globulin that needs to be administered as soon as possible after the bite to prevent the virus from spreading throughout the body. Once patients are symptomatic, there is a 99% mortality rate. Being a major zoonosis, the precise and rapid diagnosis of rabies is important for early treatment, effective prevention, and control measures.[17–23]

Current diagnostics

Traditional sellers staining test (SST) and histopathological methods are still in use for the diagnosis of rabies. Direct immunofluorescent test (dFAT) (an antibody-based test) is the reference standard test for diagnosis of rabies in fresh brain tissues of dog, as recommended by both the World Organization for Animal Health (OIE) and the WHO. The mouse inoculation test (MIT) and PCR are superior methods that are also used for routine diagnosis. While the CDC has developed a PCR-based test for rabies, its use is not suitable in resource-limited settings.[20]

PoC test

There are multiple immunological PoC tests for dogs that was developed for post-mortem brain and saliva samples.[21,24,25] Commercially available tests include Vet-o-Rabies Ag, Antigen Rapid Rabies Ag test Kit, Quicking Pet Rapid Test, Rabies Virus Ag test and VET Rabies Antigen Rapid Test.

Implementation need

The major challenge for rabies diagnosis is the use of invasive samples such as post-mortem brain tissue samples. A non-invasive sample collection method is urgently needed for use ante-mortem. Hence, there is an urgent need for the identification of circulating biomarkers. A field-deployable test for humans, that is suitable for use in primary healthcare centres, is needed for the identification of novel circulating biomarkers for rabies.

**Chromoblastomycosis**

Chromoblastomycosis (CBM) is a chronic fungal dermatosis of the skin and subcutaneous tissue caused by species of melanized fungi of the family *Herpotrichiellaceae*, which is present in soil, plants, decomposing wool, and decomposing organic matter. CBM is usually an occupational related disease that is predominant among agricultural workers, miners, and woodsmen aged between 20 to 60 years. It causes lesions on the limbs, face and neck with no direct evidence of human‑to-human or animal‑to‑human transmission. The infection starts as etiologic (disease-causing) agents enter the body through punctured wounds. While the initial lesion is similar to a small pink rash, over time (~1 week) it develops into a polymorphic clinical appearance simulating various infectious and non‑infectious diseases. The exact burden of this group of diseases is unknown.[26] CBM is a debilitating disease that is difficult to treat, and often leads to secondary bacterial infections, which poses a therapeutic challenge to clinicians. The treatment also varies in accordance to the severity of the disease and the patient’s immune response ranging from chemotherapy to combination therapy. Severe lesions respond slowly and are sometimes resistant to anti-fungal drugs. Oral antifungal therapy is not effective and must be prolonged for months or years. CBM has been reported to co-exist with other diseases including osteomyelitis, paracoccidioidomycosis, leishmaniasis, and leprosy. Co-infections reduce the efficacy of the treatment and result in prolonged sickness.[27]

Current diagnostics

Diagnosis of CBM is based on direct microscopic examination (wet mount), followed by culturing of the biopsy to determine the most effective treatment options. Growth of fungi can take up to 6 weeks, and although the genus can be identified from culture, further species determination requires DNA sequencing methods. Recently, molecular diagnostics using PCR targeting ribosomal DNA of *Fonsecaea* species and a specific DNA segment for the identification of *Cladophialohora carrionii* have been developed as a laboratory-based test. An ELISA assay using the *C. carrionii* antigen (AgSPP) has also been developed, but this is still not available in the endemic regions. The current diagnosis requires trained personnel and an equipped laboratory, and new rapid diagnostic tests are urgently needed.[27,28]

PoC test

There are currently no commercial PoC tests for CMB.

Implementation need

A PoC test with the capabilities to determine species specificity is required as therapeutic success is directly related to the causative agent.[29] **Cutaneous leishmaniasis**

Cutaneous leishmaniasis (CL) is the most common form of leishmaniasis. Globally, leishmaniasis collectively accounts for 981,000 DALYs.[30] Symptoms include debilitating skin lesions (ulcers) that leave lifelong scars, serious disability, and stigma due to mutilation. CL does not have a specific endemic area. However, in 2018, 85 % of new cases occur in Afghanistan, Algeria, Bolivia, Brazil, Colombia, Iran, Iraq, Pakistan, the Syrian Arab Republic and Tunisia. It is estimated that between 600 000 to 1 million new cases occur worldwide annually.[2] CL is treated with pentavalent antimony (e.g., sodium stibogluconate). Other medications used for treatment include amphotericin B, pentamidine isethionate, paromomycin, and antifungals.[31]

#### Current diagnostics

CL is detected by combining clinical symptoms with microscopic identification of the pathogen in Giemsa's stained skin scrapings or needle aspirates. Sample collection is often an invasive procedure and both diagnostic methods require medical expertise and are labour intensive. Furthermore, this microscopic examination often has low sensitivity. Thus, nucleic acid amplification tests (NAATs), such as PCR or nucleic acid sequence-based amplification (NASBA), which have increased sensitivity, have been developed. However, these nucleic-acid-based methods are challenging to implement in resource-limited endemic regions due to high costs and lack of resources.[32]

#### PoC test

There are three PoC tests for the diagnosis of CL. CL Detect™ Rapid Test is an immunological PoC test that detects the *Leishmania* antigens in ulcerative skin lesions. However, this test lacks specificity which results in a high number of false positive test results. The Loopamp™ *Leishmania* Detection Kit is an isothermal loop-mediated NAAT that amplifies the conserved region in the 18S rRNA of *Leishmania* and a specific sequence of the kinetoplast DNA of *Leishmania donovani.* The Loopamp is not a true PoC, as it requires DNA extraction prior to testing, but its relative simplicity makes it a possibility as a 'near'-PoC diagnostics test.[32] The palmPCR, which is a hand-held battery-operated device that targets the conserved region in the kinetoplast DNA of *Leishmania*, works very well, but it requires a trained clinician.[33–35]

#### Implementation need

While there are already several PoC devices for CL available with moderately good specificity and sensitivity, these tests need to be validated in the field. Field-deployable sample preparation is required to detect early CL infections in resource-limited settings.

**Mycetoma**

Mycetoma is a chronic, debilitating, and socially stigmatizing skin disease that is caused by more than 56 different microorganisms, bacteria (actinomycotic) and fungi (eumycotic) which reside in soil. Actinomycotic bacterial species such as *Streptomyces somaliensis, Actinomadura madurae, Actinomadura pelletieri, Nocardia brasiliensis* and *Nocardia asteroides*, and eumycotic fungal species like *Madurella mycetomatis, Madurella grisea, Pseudoallescheria boydii* and *Leptosphaeria senegalensis* are common causal agents for Mycetoma. The infection affects the feet, upper extremities, and back. Though there are no reports on human-to-human transmission or animal reservoirs (notably, it also infects animals), the disease remains endemic in certain regions due to shared environmental factors and occupations such as farming. Mycetoma is endemic in many tropical and sub-tropical countries constituting a ‘Mycetoma belt’. Mycetoma is also associated with poor personal hygiene, unavailability of protective clothing and shoes, and weaker immunity of hosts. Although mycetoma is uncommon among females, more active and aggressive forms of this disease have been observed during pregnancy possibly due to hormonal effects and suppressed immune response during pregnancy.[36,37] Accurate data on its incidence and prevalence are not available. Available antifungal drugs are ineffective, toxic, expensive, and unavailable in endemic regions, and anti-bacterial therapies require uninterrupted administration to avoid the development of resistance. Treatment is more successful in actinomycetoma (bacterial) than eumycetoma (fungal), and requires a holistic approach comprising antimicrobials, surgery, and rehabilitation.[38]

Current diagnostics

Clinical presentation of both actinomycotic and eumycotic mycetoma is identical and their differentiation is therefore required for proper case management. The time from the onset of the initial infection to seeking healthcare ranges from 3 months to 50 years, and a diagnostic delay can amount to 15 years due to symptoms that are similar to other prevalent diseases which causes misdiagnoses.[39] Thus, case management requires a detailed understanding of other infectious diseases that mimic mycetoma, including elephantiasis, yaws, chronic bacterial osteomyelitis, and other fungal diseases, and non-infectious diseases such as tumors.[37,39] Clinical diagnosis requires equipped facilities and trained personnel. Imaging procedures including ultrasound, radiography, CT and MRI scans are needed. Identification of the causal agent is also crucial to guide the treatment procedure and therefore, fine needle aspiration for extraction of grains for microscopic staining and histopathology are also employed. Molecular diagnostics using 16S rRNA and sequencing, PCR, RFLP, LAMP, and RCA are new methods that are being developed. Immunological tests have also been developed, including immunoblots and ELISA.[37,39] Existing diagnostic methods are inadequate and cannot be used as PoC tests.

PoC test

There are currently no commercial PoC tests for Mycetoma. [40]

Implementation need

Sensitive and species-specific immunological tests or nucleic-acid-based PoC tests with the possibility to function as test-of-cure are required for case detection and to monitor therapeutic outcomes, respectively.

#### **Human African Trypanosomiasis (gambiense )**

Human African trypanosomiasis (HAT) (sleeping sickness), is a parasitic protozoan disease caused by two species from the genus *Trypanosoma*: *Trypanosoma brucei gambiense* (*T. b. gambiense,* gHAT*;*98% cases) and *Trypanosoma brucei rhodesiense* (*T. b. rhodesiense,* rHAT, 2% cases, described later) Infection occurs after a bite from the vector Tsetse fly that has fed off other infected individuals. Infection could also be congenital, through other bloodsucking vectors, accidental infection in a laboratory or sexual transmission, although this is uncommon. HAT has two stages: the haemo‑lymphatic and neurological/meningo-encephalic stage. First, the parasite moves to the subcutaneous tissue and the blood and lymph vessels where it starts multiplying causing fever, headache and enlarged lymph nodes. Later, the parasites cross the blood-brain barrier and infect the central nervous system, where they cause a change in behaviour, confusion, sensory disturbances, and disturbance of the sleep cycle. HAT is fatal if left untreated. gHAT involves a long haemolymphatic stage, it often remains undiagnosed due to the generic symptoms. After several months or even years, gHAT progresses to the neurological stage. Parasitaemia remain very low throughout both stages, which makes it difficult to diagnose gHAT from body fluid samples.[2,41] Treatment for both infective species includes pentamidine, suramin, melarsoprol and eflornithine.[42] Globally, ~ 70 million individuals are at risk, mostly in resource limited settings in sub-Saharan Africa. In 2016, HAT contributed 128.4 thousand DALYs, and ranked 6th in number of deaths caused by NTDs. HAT has been a priority for the WHO and the number of reported infections dropped for the first time below 1000 in 2018. ref. [41]

Current diagnostics

Diagnosis of HAT relies on the detection of antibodies using immunological tests, parasite detection (microscopy), and stage diagnosis to determine the type of treatment that should be administered.^47^ Due to low parasitaemia levels of *T. b. gambiense*, detection mostly depends on immunological screening. The most common immunological test is the Card-Agglutination Trypanosomiasis Test, which is an antibody test that identifies the LiTat 1.3 gene present in *T. b. gambiense.* Other diagnostic techniques include Lymph Node Examination, Mini Anion Exchange Centrifugation Technique and Capillary Tube Centrifugation. Techniques that are based on staining the parasites with fluorescent dyes and UV visualisation can also be applied to diagnose gHAT. A technique called OC-PCR, wherein PCR is coupled to oligochromatography, has been developed as a proof-of-principle study. LAMP and NASBA show great potential, as it has very high specificity. However, these nucleic-acid-based tests are not commercially available.[43–45]

#### PoC test

SD BIOLINE HAT (developed by FIND and Standard Diagnostics) is a serological PoC lateral flow test for gHAT. SD BIOLOINE HAT detects antibodies that bind to the LiTat 1.5 and LiTat 1.3 antigens. This test was deployed in 2014 in endemic regions.[46] In 2018, a new version of this test was introduced (SD BIOLINE 2.0) using recombinant antigens.[47] A rapid serodiagnostic PoC test called HAT Sero *K*-SeT is also based on LiTat 1.5 antigens, and showed a comparable sensitivity and specificity to the CATT.[48] Other lateral flow assays based on multiple antigens have been reported to detect gHAT, but these tests are not commercially available yet. [49]

#### Implementation need

gHAT needs to be diagnosed early to avoid progression into the neurological stage, for which only complicated, risky, and expensive diagnosis and treatment are available. Screening of the population that is at risk is key to diagnose gHAT as early as possible. Therefore, a cost effective and easy to use PoC test is required with higher sensitivity and specificity than current immunological based PoC tests to do quick screenings of whole populations. FIND is actively developing and evaluating such tests. Test-of-cure tests are also needed to replace the cumbersome and invasive lumbar puncture, and to gauge efficacy of treat­ment.

**Buruli ulcer**

Buruli ulcer is a disabling and stigmatizing disease that is caused by *Mycobacterium ulcerans (M. ulcerans)*. It is the third most common mycobacterial disease after tuberculosis and leprosy. The mode of bacterial transmission is currently unknown. Infection with *M. ulcerans* causes ulcers that affect the skin, soft tissues and bone, resulting in a chronic debilitating illness that persists throughout the infected persons life-time. The initial symptoms are painless dermal papillae or sub-cutaneous nodule that are common to many skin NTDs, and thus diagnoses are often delayed, resulting in deteriorating patient conditions. Buruli ulcer is endemic across the global south affecting as many as 33 countries in Africa, Latin America, Australia and Japan.[2] Despite the availability of effective treatment *i.e*., a prolonged course of a combination of antibiotics (typically rifampin injections), ~2700 new cases were reported in 2018. Ref[50] Once the disease has progressed, treatment relies exclusively on surgery to remove the affected regions, including excision, skin grafting and possibly limb amputations. Disease management is difficult with HIV co‑infection and other co‑existing endemic NTDs in the tropical regions.[51]

Current diagnostics

Current diagnostic practices are conducted at the primary healthcare level or one level up, and thus require trained personnel and sophisticated instrumentation. Conventional methods for diagnoses include empirical clinical methods and microscopy, such as fluorescence microscopy using Auramine O staining and light microscopy using Ziehl‑Neelsen staining. Other methods including histopathology, bacterial culture methods, enzyme linked immunosorbent ELISA assays, and molecular diagnostics such as PCR (targeting the IS_2404_ sequence) and fluorescent thin‑layer chromatography (f-TLC) are also performed based on availability. In 2018, along with WHO, the FIND started a joint-project for the evaluation and implementation of f-TLC to detect the toxin mycolactone in lesions using monoclonal antibodies.[52–54]

PoC test

FIND is developing and piloting PoC tests for Buruli ulcer in endemic regions. The tests range from serological tests *i.e.*, an immunoassay targeting MUL_3720 protein, to NAATs based on LAMP and recombinase polymerase amplification (RPA).[54] The detection of 16s rRNA as a target is also possible. However, these tests may not be easily administered as a PoC test as they require a basic laboratory for sample preparation, extraction of DNA templates, and generation of isothermal conditions for the detection of *M. ulcerans* bacteria in the collected specimens.[54] A non-instrumented nucleic acid-based device is under development for providing an electricity-free incubation that utilizes a LAMP based test for the detection of *M. ulcerans* DNA, which may suit for field applications.[55]

Implementation need

There is a need for a field-deployable PoC test that can present test-of-cure, detect viable bacterium (active infection) and the development of other infections, which are critical in case of co‑infections.[52]

**Schistosomiasis**

Schistosomiasis is caused by an infection with trematode flatworms (schistosomes). *Schistosoma (S) haematobium, S. mansoni and S. japonicum* are the most common infective subspecies that cause human schistosomiasis. Schistosomes spread through fresh water that is infected with trematode larvae. After infection, the larvae settle in the liver where they mature into worms. Adult worms lay eggs in the blood vessels of the patient, and these eggs secrete antigenic glycoproteins which facilitate the passage of the eggs from the blood to into the internal organs or urinary bladder, where they induce an inflammatory response.[56] The eggs of adult schistosomes leave the human body in urine or faeces and hatch in fresh water where they penetrate snail hosts. In these hosts, the larvae develop and multiply cercariae (free-swimming infectious stage of schistosomes) that emerge from the snails and can penetrate human skin.[57] Symptoms of acute schistosomiasis are myalgia, diarrhoea, fatigue, malaise, and fever. [58]Active infections cause inflammatory reactions in tissues of the host. Schistosomiasis is widespread in the tropics and subtropics, especially in poor communities without clean water and adequate sanitation. ~700 million people are at risk of acquiring the infection as they live in endemic areas. 143 million people worldwide were infected in 2017, and approximately 2.5 million DALYs were estimated in 2016.ref[56,59] Schistosomiasis is treated with praziquantel which is effective for all schistosome species.[2]

Current diagnosis

*S*. *haematobium* eggs are excreted in urine and can be detected in can be detected by observing the sample under a microscope.[60] To increase the sensitivity, urine samples are often concentrated via filtration, sedimentation or centrifugation. In general, microscopy lacks sensitivity for all species as egg excretion is variable during the disease. The prevalence and intensity of infection with *S. mansoni* and *S. japonicum* is tested the Kato‑Katz technique that relies on microscopic analysis of stool samples.[61] This method is simple and inexpensive. However, it lacks sensitivity and cannot detect early infections.[57] Serological detection of anti-schistosome antibodies is also used. However, the specificity of the method is sub-optimal, and the serological tests cannot distinguish between *Schistosoma* species or between active and inactive schistosomiasis.[60] In-high resource settings, NAATs such as PCR can be used to detect the disease in serum samples to distinguish between different *Schistosoma* strains and are highly specific. In endemic settings, PCR on stool or urine is occasionally used for epidemiological studies (certainly not for routine diagnostics).[62]

PoC test

A PoC-CCA test (antigen detection) detects circulating cathodic antigen (CCA) in serum or urine.[60] This specific assay can detect *S. japonicum* and *S. mansoni* but not *S. haematobium.*[63,64] A rapid immune‑chromatographic test with higher sensitivity *to S. haematobium* in under development.[65] A smart diagnostic device known as the Schistoscope is also under development.[66] A field-deployable upconverting particle lateral flow (UCP-LF) assay for the detection of circulating anodic antigen (CAA) in serum or urine is currently under development.[12]

Implementation need

The UCP-LF CAA test is currently undergoing field studies. This antigen-based test or nucleic-acid-based PoC test would contribute to specific and early detection. Detection of *S. haematobium* infection for mapping in low prevalence areas is also needed.

**Chagas disease**

Chagas disease (CD, also known as American trypanosomiasis) is a vector-borne disease that is caused by the parasitic protozoan, *Trypanosoma cruzi,* after the bite of an infected triatomine bug. *T. cruzi* is spread through mucous membranes (eyes, mouth) and breaks in the skin (such as a wound). The dominant vectors for human transmission stem from the genera *Triatoma, Rhodnius* and *Panstrongylus*. Although CD is mainly a vector-borne disease, other forms of transmission such as congenital transmission, through blood transfusion, organ transplantation, and the consumption of contaminated uncooked food have caused the disease to spread worldwide.^2^ During the acute phase of the disease, patients are either asymptomatic or develop mild generic symptoms such as a fever, headache, a rash, or vomiting. Approximately 90 days after infection, patients become asymptomatic and this marks the chronic phase, during which ~30% of the patients develop symptoms, such as cardiac or gastrointestinal complications. Without treatment, the chronic phase can last from decades to an entire lifetime, while chronic cardiomyopathy can be fatal. Globally, CD is estimated to affect 8 million people and is responsible for 219,000 DALYs.[67–69] CD is treated with the antiparasitic drugs nifurtimox and benznidazole.

Current diagnostics

During the acute phase, microscopic examination of a blood smear can be used to identify CD due to the high parasitaemia. During the chronic phase, the parasitaemia decreases and microscopy results cannot be used to confirm a CD diagnosis. A serological test for *T. cruzi* antibodies is often used to diagnose CD. Serological tests also include complement fixation, indirect immunofluorescence assays and ELISA. Other less-sensitive diagnostic parasitological tests have been developed, including xenodiagnoses and haemoculture. Detection of parasitic DNA through PCR amplification of the minicircle fragment of the kinetoplast DNA and a 188 bp satellite sequence of nuclear *T. cruzi* DNA can also be used to detect CD. However, all these diagnostic tests require laboratory expertise and are either expensive or labour intensive.[70,71]

PoC test

There are multiple PoC immunochromatographic tests commercially available. However, only a number of these show sufficient specificity and sensitivity and are easy enough to use, but confirmation of test results in a laboratory is recommended in practice.[72] Single-use immunochromatographic tests such as Trypanasoma Detect are commercially available for the detection of antibodies against *T.cruzi*. There are proof-of-principle studies on PoC tests, such as the serological PATH-Lemos rapid assay and TESA (Trypanosomal Excreted/Secreted Antigens lateral flow test). However, these are not commercially available yet. [73,74]

Implementation need

A PoC test for early detection of congenital  transmission, rapid assessment of treatment efficiency or failure, indication/prediction of disease progression and direct parasite typification in clinical samples are urgently needed.[2,75] **Visceral leishmaniasis**

Visceral leishmaniasis, also called kala-azar (black fever), is a fatal form of leishmaniasis (described above in the page for cutaneous leishmaniasis) if left untreated. Symptoms include fever, weight loss, an enlarged spleen and liver, and severe anaemia. VL does not have a specific endemic area, but 95% of all new cases are in Brazil, China, Ethiopia, India, Iraq, Kenya, Nepal, Somalia, South Sudan and Sudan. After treatment, VL can develop into Post-kala-azar dermal leishmaniasis (PKDL), which becomes a reservoir for the parasites. PKDL usually occurs 6 months to several years after a VL patient is treated.[76,77] VL can treated with liposomal amphotericin B (AmBisome^®^), which is administered by intravenous infusion.[78] However, due to high costs and/or unavailability of AmBisome^®^, miltefosine or pentostam can be used to treat VL.

#### Current diagnostics

VL is generally diagnosed by microscopic confirmation of the parasite in bone marrow, spleen or lymph node aspirates. This requires medical expertise and often involves an invasive procedure.[79] The direct agglutination test (DAT) has been developed, which detects the presence of antibodies against the *Leishmania* parasite. However, a trained clinician is required to administer the test and results are subjectively interpreted which leads to inter-reader variation.[80–82]

#### PoC test

The IT-LEISH Kit is a prominent immunological test that detects antibodies against the rK39 antigen.[83] This test has been widely used in India, but it fails to reliably detect VL in Africa. Other immunological tests have also been developed to detect the rK28 antigen.[84] These serological tests can detect rK39-antibodies and rK28-antibodies. However, these tests fail to distinguish between current and past infections.[85–87] Another PoC test is the duplex visceral leishmaniasis lateral flow device (VL-LFD) based on a laser-patterned microfluidic assay that detects two recombinant *Leishmania* antigens: *β-tubulin* and *LiHyp1*. However, this test is not commercially available.[88] Also, the *Leishmania* Ab Rapid Test and the Kalazar detect Rapid Test are commercially available, but these are only able to detect *L. donovani* infections and should therefore always be combined with additional testing.[89] There is a mobile suitcase that has been developed for PoC diagnostics,[90] however it is not routinely used in endemic settings.

#### Implementation need

The rK39 rapid immunochromatographic test is not effective in east Africa. Field validation of the mobile suitcase and VL-LFD are required.[90] A field-deployable nucleic-acid-based test (test-of-cure) is crucial for the diagnosis of post‑kala‑azar (PKDL), and it would improve the prompt diagnosis of VL in resource-limited endemic regions.[91]

**Lymphatic filariasis**

Lymphatic filariasis (LF, also known as elephantiasis) is caused by an infection with one of three nematode species (parasitic worms) from the family *Filarioidea.* Infection, with *Wuchereria bancrofti* accounting for 90% of the infections, and *Brugia malayi* and *Brugia timori* accounting for the remaining 10%*.* The parasites are transmitted to humans by various mosquito species. LF affects the lymphatic vessels and immune system of the patient. When infected with the parasite, the worms settle in dilated nests within the lymphatic vessels where they produce millions of microfilaria (larvae) that migrate to the bloodstream. The parasite can then enter an intermediate host, the mosquito, which is the start of a new cycle of infection.[92] While the majority of infected people are asymptomatic, the disease can cause long-term chronic infections. Asymptomatic patients still get lymphatic and kidney damage. This disease is commonly found in South-East Asia and Africa. More than 40 million people have the clinical manifestations such as lymphoedema of the limbs (elephantiasis).[92] Globally, approximately 120 million people are infected with LF, wherein one-third of the infected have the clinical manifestations such as elephantiasis. Diethylcarbamazine (DEC) treatment is effective against LF, however, DEC cannot be used in regions where onchocerciasis and loiasis are present. LF can be treated with MDA of ivermectin, especially in settings where onchocerciasis is present.[93]

Current diagnostics

The reference standard diagnosis of LF relies on the detection of microfilariae in blood samples by microscopic examination. Routine laboratory assays such as ELISA can also be used to detect elevated levels of antifilarial IgG4 antibodies.[94,95]

PoC test

There are multiple PoC tests for lymphatic filariasis that are commercially available. A Alere filariasis strip test detects antigens of *W. bancrofti.*[95,96] Other commercial tests include rapid anti-filarial antigen-based tests such as *Brugia* Rapid and SD BIOLOINE Lymphatic filariasis.

Implementation need

Current PoC tests that detect antigens of *W. bancrofti* have cross-reactivity with antigens of the infectious disease loiasis (African eye worm), which has been recently recognized as a serious obstacle to eliminate LF in loiasis-endemic areas.[96] A PoC test without cross‑reactivity with loiasis could contribute to the elimination of LF. Novel biomarker identification is also required to facilitate the development of multiplexed tests for LF and loiasis.

**Chikungunya**

Chikungunya is a viral disease that is caused by an alphavirus in the family *Togaviridae*, which is transmitted by the bite of female mosquitos *Aedes aegypti* and *Aedes albopictus*. This disease shows similar transmission and symptomatic patterns as dengue and malaria infections. Chikungunya can also exhibit latrogenic transmission via blood transfusions or organ transfusions (liver, kidney etc.). Since the chikungunya virus has been detected in semen 30 days after symptoms onset, sexual transmission is also a possibility, but no evidence for this has been reported so far. Chikungunya is an emerging seasonal febrile illness that is characterized by intense joint pain and it can cause lifelong effects such as arthritis, chronic inflammatory rheumatism, and neurological manifestations.[97] During a 2006 epidemic in India, the burden was estimated to be 25,600 DALYs lost, with an overall burden of ~ 45 DALYs per million people. A chikungunya epidemic across the Americas in the period of 2013-2015 had a total of over 39.9 million cases imposing a burden of over 23.8 million (DALYs) lost.[98] Disease management is difficult during pregnancy as it can impact fetal development, and cause neonatal infections resulting in severe acute cases of microcephaly and cerebral palsy. With an unprecedented increase in chikungunya spread across more than 60 countries, there is an urgent need to combat this disease. Although vaccination programs have been initiated, eradication is still dependent on early diagnoses for effective treatment. There is not specific treatment for chikungunya, but the symptoms can be alleviated with medication. [99]

Current diagnostics

Laboratory based tests are used to diagnose chikungunya in serum or plasma to detect viral nucleic acids or virus-specific IgM and neutralizing antibodies at 5 to 7 days after symptom onset. Specific chikungunya virus IgG can be detected post treatment for several years. There are reports on false positives due to cross-reactions with other alphaviruses. RT-PCR is used to detect the chikungunya virus and is performed 7 days after the onset of symptoms. Viral cultures in biosafety level 3 laboratories are also used to detect chikungunya. The clinical challenge lies in the inability to distinguish chikungunya from dengue as both diseases usually occur during the same seasons. Serological tests, including ELISA, can confirm the presence of antibodies. While RT-PCR methods are available to detect the virus in blood samples, their sensitivity varies and they are not suitable for endemic resource-limited settings.[97]

PoC test

An antibody screening test (antigen-based) for chikungunya has been developed that detects IgM antibodies in a solid-phase immunochromatographic assay (called SD BIOLINE Chikungunya), and there is the Chikungunya IgM Combo Rapid Test (CE) that is commercially available*.* [100]

Implementation need

There is an urgent need for a field-deployable PoC test for chikungunya that does not have any cross reactivity with dengue. A multiplexed test with dengue would also greatly improve the diagnosis and treatment of chikungunya. Novel biomarker identification would facilitate the development of a multiplexed text that does not have any cross reactivity with dengue. [101]

**Scabies**

Scabies is a curable skin infestation that is caused by a microscopic mite known as *Sarcoptes scabiei* var. *hominis.*[102,103] Scabies is highly contagious, and is transmitted from person-to-person through close skin contact. Once infected, the female mite settles in the skin and lays eggs, thus triggering an immune response that causes intense itching and a rash. The skin is then exposed to bacterial infections that can cause severe soft-tissue infections, septicaemia, kidney disease, and rheumatic fever. Approximately 455 million people are infected by scabies, causing ~5,6 million DALYs.[30] Although people of all ages can become infected, scabies mostly affects children. Outbreaks in residential and nursing homes for the elderly are common in high-resource settings.[103] Immunocompromised people, such as HIV positive people, are more prone to develop crusted scabies.[102] MDA using oral ivermectin and topical scabicides, is an effective treatment strategy. Topical scabicides include permethrin, benzyl benzoate, malathion, and sulphur ointment.

Current diagnostics

Scabies is diagnosed empirically, using the patient’s history and a physical examination. Scabies presents with visible symptoms within 2-6 weeks of the initial infestation. Common presenting lesions are papules, pustules, and nodules. Secondary bacterial infections with *Staphylococcus* or *Streptococcus*, as a result of scratching, are often misleading. Definitive diagnosis relies on microscopy to identify mites or eggs from skin scrapings. However, treatment is often started without microscopic confirmation. Serological methods have not been successful in detecting human infections.^102^ DNA libraries of *S. scabiei* var. *hominis* have been constructed for epidemiological studies, but commercial molecular diagnostic tests have not been developed.[104] Essentially, there is no standard diagnostic procedure which leads to misdiagnoses.[103,105] Critical action is required to improve individual diagnosis as misdiagnosis and delayed treatment are the main challenges to eradicating scabies.[106]

PoC test

There are two PoC tests for scabies, the burrow ink test and the handheld dermatoscopy microscopy tool.[104] The burrow ink test is a simple non-invasive test than can be used to screen a large number of patients. A fountain pen is gently rubbed onto a suspected lesion. Excess ink is wiped off with an alcohol swab, making the infected site (burrow) visible with a wavy ink-filled line where the mites have tunnelled into the skin. The handheld dermatoscopy tool is accurate but it is expensive and requires trained users.[107]

Implementation need

There is a need for diagnostic methods that are standardized, and circumvent complex sample preparation, and that can take place at the PoC. This would be beneficial in reducing scabies, predominantly by preventing misdiagnosis and delayed treatment.[103]

**Onchocerciasis**

Onchocerciasis, caused by a parasitic worm *Onchocerca volvulus*, is a one of the NTDs that is slowly approaching elimination.[108] It is also known as river blindness as it causes an eye and skin disease among the inhabitants living nearby rivers that breed Simulium black flies, which transfer adult female worms to a human body during a blood meal. A single adult female worm produces thousands of microfilariae (larvae) which migrate into the skin and eyes.[109] The death of these microfilariae results in the release of toxic chemicals into the eyes and skin, eventually causing blindness and disfigured skin. Onchocerciasis has been endemic within Africa and the Americas with approximately 21 million cases in 2017 and approximately 205 million DALYs in 2017. ref[110] MDA of Ivermectin and vector control strategies facilitated the containment of the disease, bringing it now close to the elimination of transmission.[111]

Current diagnostics

While microscopic examination of skin biopsies is the reference standard for the diagnosis of onchocerciasis, molecular diagnostics using PCR are performed as a test-of-cure. Real-time PCR and LAMP assays are also performed, as they can provide a rapid colorimetric readout to detect even a single parasite.[112] Immunological diagnoses using ELISA have been the most economical diagnostic tests, though they still require cold chain, equipped laboratory, and trained personnel, thus making it unsuitable for diagnosis in endemic resource-limited settings.

PoC test

There is a SD BIOLINE biplex PoC test for the detection of Onchocerca volvulus and Wuchereria bancrofti antigens, and a SD BIOLINE Onchocerciasis IgG4 test that detects antibodies in a sample in response to OV16 antigens. [113]

Implementation need

Current PoC tests that detect *W. bancrofti* antigens have cross-reactivity with antigens of the infectious disease loiasis. A field-deployable PoC test that does not have cross reactivity with *Loa loa* antigens is required, as co-infection with onchocerciasis and loiasis affects the treatment regimen. Novel circulating biomarker identification is required to identify specific biomarkers that do not cross react. A confirmatory test-of-cure PoC would facilitate the eradication, and surveillance thereafter, of this disease.

**Human African trypanosomiasis (*rhodesiense*)**

HAT caused by *T. b. rhodesiense* (rHAT) accounts for 2% of all reported cases and occurs in 13 endemic countries in eastern sub‑Saharan Africa.[2,41] Similar to gHAT (described above in the page for gHAT), rHAT also spreads through the bite from a Tsetse fly (vector) and causes an acute infection. First signs and symptoms are observed a few months or weeks after infection. The disease develops rapidly and invades the central nervous system. Only Uganda presents both forms of the disease, but in separate zones. Treatment for both infective species includes pentamidine, suramin, melarsoprol and eflornithine.[42]

#### Current diagnostics

Parasite detection (microscopy) is a valuable diagnostic method for rHAT as the acute phase corresponds with high parasitaemia levels. Thick and thin film stained with Giemsa is the commonly used method for rHAT diagnosis.[114] Other less popular yet effective diagnostic techniques include Lymph Node Examination and Capillary Tube Centrifugation. Staining of the parasite with UV fluorescent dye can also be applied to diagnosing rHAT. A lumbar puncture is used to stage rHAT *i.e.,* to determine if the parasite has reached the central nervous system. The nucleic acid-based approaches for rHAT including a technique called OC-PCR, wherein PCR is coupled to oligochromatography, has been developed as a proof-of-principle study. LAMP and NASBA show great potential, as it has very high specificity. However, these nucleic-acid-based tests are not commercially available. [43–45]

#### PoC test

There are currently no commercial PoC tests for rHAT.

#### Implementation need

The identification of novel circulating biomarkers for rHAT is required to ensure the timely diagnosis of this disease in endemic resource-limited settings. A field-deployable nucleic-acid-based test would facilitate the species-specific detection of rHAT, which will aid effective treatment.

**Snakebite envenoming**

Snakebite envenoming (SBE) is caused by the injection of venom into the patient after the bite of a venomous snake, while certain snake species spray venom directly into the patient’s eyes.[115] Snakebites can be fatal as the toxins can cause paralysis that prevent breathing, or bleeding disorders that can lead to fatal haemorrhaging.[116] Other SBE effects include irreversible kidney failure and tissue damage that can lead to permanent disability or limb amputation. Approximately 2,7 million people are bitten by snakes with envenoming annually, causing 80,000 – 140,000 deaths, and ~6-8 million DALYs.[2] The availability of antivenom treatment (venom neutralizing antibodies) is expensive and limited in supply, and has a number of associated adverse effects, making administering antivenom risky.[116] Antivenom cannot reverse tissue damage caused, but it can neutralize active circulating venom to prevent further tissue damage.[115] A reliable diagnostic tool would alleviate the damage caused by SBE, but it is unlikely that snakebites in general can be eradicated.

Current diagnostics

Empirical diagnosis is often used to diagnose SBE. Clinical symptoms include blistering, swelling, bleeding, tissue necrosis and pain. Verbal confirmation from the patient is also used to verify SBE if the patient is lucid. When symptoms are less clear, a 20-minute whole blood clotting test is used to determine SBE. In such tests, it is essential that the glassware is standardised, and the sample volume and temperature need to be regulated and validated using serial donor samples prior to routine use. More specific molecular tests are being developed but are not considered PoC tests as they rely on laboratory-based blood coagulation analysers.[116]

PoC test

There is one commercial antibody-based diagnostic test, the Australian commonwealth serums lab snake venom detection kit.[2] The major limitation of this test is that it is a matching tool for 5 of the Australian antivenom treatments. There is also cross reactivity between venoms, which leads to false positive results. [116]

Implementation need

A PoC test is needed to detect venom-induced coagulopathy, a bleeding disorder that occurs when the bloods ability to coagulate is impaired.[116] Species specific diagnosis is not essential for effective treatment[2] as polyvalent antivenom (against different species) is produced more commonly than monovalent antivenom (against a single species). A more practical need is a region-specific multiplexed PoC test, as snake species are geographically separated.[117] The international normalized ratio (INR) is a screening method for venom-induced consumption coagulopathy after snakebites. INR currently relies on a laboratory coagulation analyser, and studies have demonstrated that the PoC INR is not reliable as it results in false negative test results.[118] Further research is required to develop a reliable PoC coagulation analyzer which would enable more reliable PoC diagnosis of venom-induced coagulopathy. **Dengue**

Analogous to chikungunya, dengue is also an acute arboviral disease that is transmitted by the bite of an infected female *Aedes* mosquito. Non-vector transmissions through blood transfusions, organ transplantations, mucosal splashes have also been reported. Although the virus has been found in semen and vaginal secretions, no confirmed cases of sexual transmission have been reported. While vertical transmission from a viraemic mother to her foetus through placental transmission is plausible, increasing the risk of preterm birth or fetal death, transmission to infants though breast feeding has been ruled out. Dengue infections occurs over a period of 3-4 weeks until recovery. This disease presents in different phases ranging from a febrile phase with a headache, malaise and general body pain from the onset of the disease, to the critical phase wherein plasma leakage can lead to the respiratory distress, persistent vomiting, abdominal pain, and secondary infections. The pathogenicity in severe cases is due to cross-reactivity of antibodies (antibody-dependent enhancement), resulting in the greater burden of the infection to the vascular system which leads to dengue shock syndrome,[119] coagulation abnormalities, and bleeding in hepatic and neurological organs, and also renal and ocular impairment. Dengue occurs globally and results in a socioeconomic and disease burden on the healthcare facilities in tropical and sub‑tropical regions. There are approximately 400 million infections per year that amount to 3 million DALYs globally.[120] Due to international travelling of asymptomatic carriers and patients exhibiting minor symptoms, dengue has shown a rapid geographic spread and cases have been reported in temperate climates such as in USA and Europe (Croatia, France, Portugal).[2] Recent research has focused on vaccine development and transmission control measures.[119] There is not specific treatment for dengue, but the symptoms can be alleviated with medication.[121]

Current diagnostics

There are multiple laboratory-based diagnostic methods for the detection of the dengue virus, including virus isolation in cell culture, viral RNA detection by NAATs such as RT‑PCR, and the detection of viral antigens such as the NS1 antigen detection test that detects secreted proteins in blood samples. ELISA can be performed 4-5 days after the onset of initial symptoms. Since dengue IgM antibodies start to increase from day 4 onwards, peak at about days 10–14, and then decline and disappear after about 3 months, serological assays are preferred. Cross-reactivity with Zika virus is reported for all immunological assays as well as the NS1 antigen test.[119]

PoC test

There are multiple commercial immunological PoC tests, such as the SD BIOLOINE Dengue Duo (Dengue NS1 AG+IgG/IgM) which is an antibody-based (to detect IgG and IgM antibodies) and antibody-based (to detect NS1 antigens) test, and the ASSURE Dengue IgA Rapid test (antigen-based).[122–124]

Implementation need

Newly developed tests will need to be validated in the field in endemic regions. A field-deployable PoC test that does not have any cross reactivity with the Zika virus is required. Novel circulating biomarker identification is required to identify specific biomarkers that do not cross react. A multiplexed test with the Zika virus can then be developed.[106]

**Dracunculiasis**

Dracunculiasis is a disabling parasitic disease that is caused by the nematode Guinea worm *Dracunculus medinensis*. It is transmitted to people from drinking stagnant water with parasite-infected water fleas that contain the larvae of *D. medinensis*. Infected patients often immerse their limbs into water to relieve the burning sensation when the worm is emerging. The worm then releases larvae into the water, completing the circle of transmission. Animals are also infected, especially dogs. Although this disease is rarely fatal, patients become non-functional for weeks when the adult female worms emerge from the body. Surgical removal of the worm is possible in high-resource settings.[2,125] Dracunculiasis is on the verge of eradication with only 28 human cases reported in 2018 and 54 cases reported in 2019. In terms of eradication, only 7 countries remaining need to be certified as free of the disease (Angola, Chad, Ethiopia, Mali, South Sudan, Sudan, and the Democratic Republic of Congo). There is currently no treatment or vaccine to prevent this disease.[2,106] Current eradication strategies, which proved effective, involved treating bodies of water with Temephos (larvicide) to provide safe drinking water, and providing health education to increase awareness in affected communities. Topical antibiotics can be used to prevent secondary infections.

Current diagnostics

Dracunculiasis is diagnosed by clinical presentation, based on the appearance of a skin lesion with a protruding worm that can be seen with the naked eye, usually on the lower limb but also on the abdominal region. Blood tests reveal elevated eosinophilia and immunoglobulin G4 levels. Alternatively, x-rays can reveal dead calcified worms if the worms die before they exit the skin. Immunological diagnostic tests are not useful in practice as it has not been determined if they can detect prepatent infections (the period between infection with a parasite and the demonstration of the parasite in the body) – mainly due a lack of prepatent serum samples. Evidence has not been found for circulating antigens during prepatent infections. There is evidence for antibody detection during patent infections against whole-worm antigens.[126]

PoC test

There are currently no PoC tests for dracunculiasis.

Implementation need

Testing bodies of water are more useful than testing individuals as the patient symptoms are very clear. Surveillance is the key to ensure eradication certification to countries that are on the verge of eradication. There is a need for a field-based PoC test for humans to aid ongoing surveillance, for dogs to assess canine prevalence[127] and a need for a pond-side test for detecting *D. medinensis* DNA in copepods.

**Leprosy**

Leprosy is a chronic infectious disease that is caused by the bacteria *Mycobacterium leprae.* Known routes of transmission of *M. Leprae* are droplet infection via the nose and mouth, or via skin-to-skin contact. When infected, *M. leprae* patients enter the relatively asymptomatic early disease stage, and it can take 5-10 years before clinical symptoms arise. These symptoms are characterized by hypopigmented macules, which are common characteristics of other skin diseases and which rarely leads to the diagnosis of leprosy.[128,129] If left untreated, the disease may develop into a permanent stage of leprosy. The WHO classifies permanent leprosy in two clinical variants: paucibacillary leprosy where the immune system of the patient is sufficient, and multibacillary leprosy where the immune system of the patient is poor. This classification is useful for diagnosis and treatment in the field. The permanent stage is classified by skin lesions, damage to the nervous system, and eventually disfigured mutilations of limbs.[129] A total of 176,176 patients from 138 countries were treated for leprosy in 2015. The highest incidence of patients was seen in India, Brazil, and Indonesia. The DALY was estimated to be 2,07 million in 2015. ref[130] Leprosy is treated with multidrug therapy that contains three different antibiotics.[129]

Current diagnostics

Diagnosis of leprosy is difficult as it does not have unique characteristics. Leprosy is clinically diagnosed if patients show two out of three typical signs, defined as loss of sensation in a skin lesion, enlarged peripheral nerve, and positive detection of bacilli in skin smears from the lesion. Laboratory-based tests have difficulties in detecting certain forms of leprosy, and thus clinical diagnosis is still necessary. Microscopic detection of *M. leprae* infection is done by observing stained bacteria in lymph fluid of skin lesions using light microscopy. ELISA, lateral flow assays have low accuracy. Therefore, the most sensitive laboratory diagnostic tool for *M. leprae* is PCR which targets *M. leprae* specific genes.[128,129] Where the sensitivity and specificity of PCR tests is close to 100% for multibacillary leprosy, PCR only has sub-optimal sensitivity and specificity for paucibacillary leprosy.[131]

**PoC test**

There are currently no PoC tests that are commercially available for the diagnosis of leprosy. However, a recent study showed promising results for a lateral flow assay that is based on three new specific biomarkers for leprosy (ApoA1, IL-1Ra, S100A12).[132]

**Implementation Need**

Newly developed tests, such as the lateral flow assay discussed above, will need to be validated in the field before it becomes commercially available. Diagnosis of infection and screening for potential disease is needed to control disease transmission across populations.

**Soil-transmitted helminthiases**

Soil-transmitted helminthiases (STHs) are a group of intestinal parasites that include *Ascaris lumbricoides*, *Strongyloides stercoralis*, *Trichuris trichiura*, and hookworms *Necator americanus* and *Ancylostoma duodenale*. STHs are transmitted, depending on the species, either by eggs or larvae present in human faeces, and are highly prevalent in many tropical areas with poor sanitation. STHs cause aneamia, malnutrition, impaired growth and cognitive development, abdominal pain, diarrhoea, and hyper infection syndrome. The latter can be fatal. There were 1.5 billion people infected with STHs in 2003, ~6,300 deaths in 2016 and ~3.5 billion DALYs in 2016. STHs are widely distributed in tropical and subtropical regions, with high infection rates in Sub-Saharan Africa, the Americas, China, and South East Asia. Deworming is recommended by the WHO as a preventive measure for people who are at risk. Deworming treatment includes albendazole and mebendazole for *A lumbricoides*, *T. trichiura*, and hookworms, and ivermectin for *S. stercoralis.* Although the deworming treatment is very effective, the availability of deworming medication is limited in heavily burdened resource-limited regions.[2]

Current diagnostics

STHs are currently diagnosed by microscopic visualization of parasite eggs or larvae. The Kato-Katz method is the reference diagnostic method that relies on microscopic analysis of stool samples. However, this method has low sensitivity when the worm burden is low, and it requires time, specialized skills and equipment, making it less suitable for daily use in health clinics inf resource-limited settings. Currently, DNA detection assays, such as PCR, of parasite DNA in faeces is widely used for epidemiology studies but not for routine diagnosis as it is more expensive than the deworming treatment which is administered on a large scale.[3] Other microscopy methods include Mini-FLOTAC and the Baermann technique. ELISA is also preferred for diagnosing strongyloidiasis.[133]

PoC test

There is a proof-of-principle study for a smart microscopy tool called Kankanet, which is a smartphone equipped with a USB video class microscope attachment. Kankanet is set to recognise *Ascaris lumbricoides, Trichuris trichiura* and hookworms.[134] However, it has been shown that Kankanet only works well for *Ascaris lumbricoides* when compared to the Kato-Katz method using faecal samples. LAMP based assays and colorimetric isothermal assay (SmartAmp2) to identify hookworm (*N. americanus*) have been developed for screening.[133]

Implementation need

Research to improve the algorithms that recognize the different STH species are needed to further validate the use of smart microscopy tools. A field deployable PoC test that detects specific biomarkers would improve diagnoses in the field. Multiplexed tests would also be beneficial as infections with different STH species are common in endemic regions.[2] Standardized diagnostic procedures and guidelines are required to limit variations in prevalence for more effective epidemiology studies. A field-deployable test to detect resistance to treatment is also urgently needed.[135]

**Trachoma**

Trachoma is an eye disease that is caused by infection with the bacterium *Chlamydia trachomatis*. It is spread by personal contact (*via* hand contact, clothes, and sharing linen) and through house flies (*Muscidae*) that come in contact with discharge from the eyes or nose of an infected person. Trachoma causes trichiasis after repeated infections, which occurs when the eyelashes turn inwards and scratch the surface of the eye and permanently damage the cornea. Although trichiasis is not fatal, it is extremely painful and results in irreversible visual impairment and eventual blindness. Trichiasis is the world’s leading infectious cause of preventable blindness. Trachoma remains endemic in the global south (Sub-Saharan Africa, and South America) with 142 million people (2019) still at risk of acquiring the infection. Trachoma is treated by MDA of azithromycin, which acts to reduce the ocular reservoir of *C.trachomatis*. Improved access to sanitation to improve facial cleanliness greatly reduces the effects of trachoma. Environmental improvements, such as removal of human faeces from the environment, reduces breeding sites for the vectors (muscid flies).[2] The SAFE strategy is an effective prevention approach (Surgery for infected people, Antibiotic MDA to clear ocular *C.trachomatis,* Facial cleanliness and Environmental improvement to reduce transmission).

Current diagnostics

Trachoma is diagnosed by medical staff and trained healthcare workers. Diagnosis is done empirically with a grading system, based on visible physical symptoms. WHO provides 5 grading classifications: Trachomatous Inflammation Follicular - which mostly requires topical treatment. Trachomatous Inflammation Intense - during which topical and systemic treatments are considered. Trachomatous Scarring when scars are visible as in the tarsal conjunctiva and which may obscure tarsal blood vessels. Trachomatous Trichiasis when an individual is referred for eyelid surgery, and finally Corneal Opacity - a stage during which a person is irreversibly blind. The greatest challenge is that early infections often do not present visible symptoms. Although not used in practice much, a sample of fluid from the eye can be sent to a lab for testing. PCR or culture methods can then be used, but these methods are too expensive for use in national programmes.[136] There are numerous laboratory-based tests that are useful for low prevalence areas. *C.trachomatis* can be cultured in different cell culture systems. There are cytologic tests by Geimsa stain or direct fluorescent antibody tests as well as an enzyme immunoassay that detect antibodies against chlamydial antigens.[137] PoC tests are required to indicate when certain districts need to start MDA interventions before the infection becomes widespread.

PoC test

There is a PoC test for trachoma that detects antibodies against an antigen (pgp3) from *C.trachomatis.*[138] This test works well in the field, but is limited to advanced infections. There is a grading tool that helps healthcare workers to measure the follicle size with a graded adhesive sticker that fits on the graders thumb. [139]

Implementation need

A confirmatory (test-of-cure) PoC test that can detect acute infections is required to determine when MDA can be discontinued in an endemic region. [140]

**Yaws**

Yaws is a bacterial infection that is caused by the *Treponema pallidum* subspecies *pertenue*, which is closely related to syphilis.[141] This disease is spread by direct skin-to-skin contact with highly infectious lesions. Yaws is characterized by different stages of clinical manifestation. After a variable incubation period (between 10 and 90 days) a primary lesion appears at the patient’s infection site, which can develop into a large papilloma (ulcer). Without antibiotic treatment, the patient can develop secondary lesions in a few weeks to 2 years after the primary lesion. Yaws can develop into a chronic stage of infection which results in severe deforming bone lesions. Patients with yaws can also enter a latent stage of infection, which can only be serologically determined and will last for a lifetime. Yaws is endemic in the warm, humid tropical areas of Africa, Asia, Latin America and the Pacific Islands. The most affected group are children in poor rural areas. Approximately 80.000 cases of yaws were reported (2018) and 1.6 million DALYs (2014).ref[142,143] Treatment includes the use of two antibiotics, azithromycin or benzathine penicillin.[142]

Current diagnostics

Yaws is most commonly diagnosed by treponemal and non-treponemal immunological tests that are also used for syphilis. For treponemal tests, the *Treponema pallidum* particle agglutination assay, *Treponema pallidum* hemagglutination assay or the fluorescent treponemal antibody test are commonly used. For non-treponemal tests, the Rapid Plasma Reagin or Venereal Disease Research Laboratory assays are commonly used. Treponemal tests are highly specific but cannot distinguish between treated or non-treated Yaws. Non-Treponemal tests are not specific but can detect active infections after treatment. Thus, both immunological tests are needed for an accurate diagnosis. [142]^,^[144] Other methods that are used to detect yaws are dark-field microscopy and direct nucleic acid detection using PCR. Dark-field microscopy requires fresh samples of active lesions. Tests that are based on nucleic-acid-based detection (qualitative or quantitative PCR) and are often followed by detection with labelled probes to increase the sensitivity and specificity.[145] Nucleic-acid‑based tests can distinguish between syphilis and yaws. However, nucleic-acid-based tests are limited to well-equipped laboratories.[142] [145]

PoC test

The Dual Path Platform test (DPP^®^) can be used as a PoC test to detect both treponemal and a non-treponemal infections. DPP^®^ was tested successfully in China and later tested in resource-limited settings to determine the healthcare workers and patient’s perceptions of the test.[144,146] There is also a LAMP proof‑of‑principle test that can be applied to target a specific yaws gene in swab samples from lesions. However, this LAMP assay has not been adapted for field deployability.[147]

Implementation need

A field-deployable nucleic-acid-based PoC test is needed to distinguish between yaws and syphilis in resource-limited settings. A multiplexed test for yaws and syphilis would facilitate effective diagnoses of these diseases.

**References:**

1. Li L, Liu X, Zhou B, Zhang S, Wang G, Ma G, et al. Multiple food-borne trematodiases with profound systemic involvement: a case report and literature review. BMC Infectious Diseases. 2019;19: 526. doi:10.1186/s12879-019-4140-y

2. WHO. WHO global consultations for new roadmap on NTDs. 2019. Available: https://www.who.int/neglected_diseases/news/WHO-global-consultations-for-new-Roadmap-on-NTD/en/

3. Bergquist R, Johansen MV, Utzinger J. Diagnostic dilemmas in helminthology: what tools to use and when? Trends in Parasitology. 2009;25: 151–156. doi:10.1016/j.pt.2009.01.004

4. Mubanga C, Mwape KE, Phiri IK, Trevisan C, Zulu G, Chabala C, et al. Progress on the development of rapid diagnostic tests for foodborne neglected zoonotic helminthiases: A systematic review. Acta Tropica. 2019;194: 135–147. doi:10.1016/j.actatropica.2019.03.030

5. Monteiro KJL, Calegar DA, Group Amazonas Research, Carvalho-Costa FA, Jaeger LH. Kato-Katz thick smears as a DNA source of soil-transmitted helminths. Journal of helminthology. 2018;94: e10. doi:10.1017/s0022149x18001013

6. Sadaow L, Sanpool O, Rodpai R, Yamasaki H, Ittiprasert W, Mann VH, et al. Development of an Immunochromatographic Point-of-Care Test for Serodiagnosis of Opisthorchiasis and Clonorchiasis. The American Journal of Tropical Medicine and Hygiene. 2019;101: 1156–1160. doi:10.4269/ajtmh.19-0446

7. Fleury A, Cardenas G, Adalid-Peralta L, Fragoso G, Sciutto E. Immunopathology in Taenia solium neurocysticercosis. Mahanty S, editor. Parasite immunology. 2016;38: 147–157. doi:10.1111/pim.12299

8. Garcia HH, Nash TE, Brutto OHD. Clinical symptoms, diagnosis, and treatment of neurocysticercosis. Lancet Neurology. 2014;13: 1202–1215. doi:10.1016/s1474-4422(14)70094-8

9. WHO. WHO Estimates of the Global Burden of Foodborne Diseases. 2016.

10. Mohammad IN, Heiner DC, Miller BL, Goldberg MA, Kagan IG. Enzyme-Linked Immunosorbent-Assay for the Diagnosis of Cerebral Cysticercosis. Journal of Clinical Microbiology. 1984;20: 775–779.

11. WHO. Taenia solium taeniasis/cysticercosis diagnostic tools: report of a stakeholder meeting, Geneva, 17-18 December 2015. 2016.

12. Corstjens PLAM, Dood CJ de, Priest JW, Tanke HJ, Handali S, in PCWG. Feasibility of a lateral flow test for neurocysticercosis using novel up-converting nanomaterials and a lightweight strip analyzer. Gabriël S, editor. PLOS Neglected Tropical Diseases. 2014;8: e2944–e2944. doi:10.1371/journal.pntd.0002944

13. Tamarozzi F, Mariconti M, Covini I, Brunetti E. [Rapid diagnostic tests for the serodiagnosis of human cystic echinococcosis]. Bulletin de la Societe de pathologie exotique (1990). 2017;110: 20–30. doi:10.1007/s13149-017-0548-z

14. Otero-Abad B, Torgerson PR. A Systematic Review of the Epidemiology of Echinococcosis in Domestic and Wild Animals. Garcia HH, editor. PLOS Neglected Tropical Diseases. 2013;7. doi:10.1371/journal.pntd.0002249

15. Schipper HG, Kager PA. Diagnosis and treatment of hepatic echinococcosis: An overview. Scandinavian Journal of Gastroenterology. 2004;39: 50–55. doi:10.1080/00855920410011004

16. Tamarozzi F, Covini I, Mariconti M, Narra R, Tinelli C, Silvestri AD, et al. Comparison of the Diagnostic Accuracy of Three Rapid Tests for the Serodiagnosis of Hepatic Cystic Echinococcosis in Humans. Hirayama K, editor. PLOS Neglected Tropical Diseases. 2016;10: e0004444. doi:10.1371/journal.pntd.0004444

17. Fooks AR, Cliquet F, Finke S, Freuling C, Hemachudha T, Mani RS, et al. Rabies. Nature reviews Disease primers. 2017;3: 17091–19. doi:10.1038/nrdp.2017.91

18. Jackson AC. Rabies: a medical perspective. Revue scientifique et technique (International Office of Epizootics). 2018;37: 569–580. doi:10.20506/rst.37.2.2825

19. Moore SM. Rabies: Current Preventive Strategies. The Veterinary clinics of North America Small animal practice. 2019;49: 629–641. doi:10.1016/j.cvsm.2019.02.014

20. Singh R, Singh KP, Cherian S, Saminathan M, Kapoor S, Reddy GBM, et al. Rabies - epidemiology, pathogenesis, public health concerns and advances in diagnosis and control: a comprehensive review. The veterinary quarterly. 2017;37: 212–251. doi:10.1080/01652176.2017.1343516

21. Certoma A, Lunt RA, Vosloo W, Smith I, Colling A, Williams DT, et al. Assessment of a rabies virus rapid diagnostic test for the detection of Australian bat lyssavirus. Tropical medicine and infectious disease. 2018;3: 109. doi:10.3390/tropicalmed3040109

22. Fooks AR, Banyard AC, Horton DL, Johnson N, McElhinney LM, Jackson AC. Current status of rabies and prospects for elimination. Lancet. 2014;384: 1389–1399. doi:10.1016/s0140-6736(13)62707-5

23. Elmore SA, Chipman RB, Slate D, Huyvaert KP, VerCauteren KC, Gilbert AT. Management and modeling approaches for controlling raccoon rabies: The road to elimination. Zinsstag J, editor. PLOS Neglected Tropical Diseases. 2017;11. doi:10.1371/journal.pntd.0005249

24. Léchenne M, Naïssengar K, Lepelletier A, Alfaroukh IO, Bourhy H, Zinsstag J, et al. Validation of a Rapid Rabies Diagnostic Tool for Field Surveillance in Developing Countries. Rupprecht CE, editor. PLOS Neglected Tropical Diseases. 2016;10: e0005010. doi:10.1371/journal.pntd.0005010

25. Kang B, Oh J, Lee C, Park B-K, Park Y, Hong K, et al. Evaluation of a rapid immunodiagnostic test kit for rabies virus. Journal of virological methods. 2007;145: 30–36. doi:10.1016/j.jviromet.2007.05.005

26. Brito AC de, Bittencourt M de JS. Chromoblastomycosis: an etiological, epidemiological, clinical, diagnostic, and treatment update. Anais brasileiros de dermatologia. 2018;93: 495–506. doi:10.1590/abd1806-4841.20187321

27. Queiróz AJR, Domingos FP, Antônio JR. Chromoblastomycosis: clinical experience and review of literature. International journal of dermatology. 2018;57: 1351–1355. doi:10.1111/ijd.14185

28. Hay R, Denning DW, Bonifaz A, Queiroz-Telles F, Beer K, Bustamante B, et al. The Diagnosis of Fungal Neglected Tropical Diseases (Fungal NTDs) and the Role of Investigation and Laboratory Tests: An Expert Consensus Report. Tropical medicine and infectious disease. 2019;4: 122. doi:10.3390/tropicalmed4040122

29. Bienvenu A-L, Picot S. Mycetoma and Chromoblastomycosis: Perspective for Diagnosis Improvement Using Biomarkers. Molecules. 2020;25: 2594. doi:10.3390/molecules25112594

30. Soriano JB, Banoub JAM, Kanchan T, Yasin YJ. GBD 2017 DALYs and HALE Collaborators. Global, regional, and national disability-adjusted life-years (DALYs) for 359 diseases and injuries and healthy life expectancy (HALE) for 195 countries and territories, 1990-2017: a systematic analysis for the Global Burden of Disease Study (vol 392, pg 1859, 2018). Lancet. 2019;393: E44–E44. doi:10.1016/s0140-6736(19)31043-8

31. Markle WH, Makhoul K. Cutaneous leishmaniasis: recognition and treatment. Am Fam Physician. 2004;69: 1455–60.

32. Schallig HDFH, Hu RVP, Kent AD, Loenen M van, Menting S, Picado A, et al. Evaluation of point of care tests for the diagnosis of cutaneous leishmaniasis in Suriname. BMC Infectious Diseases. 2019;19: 25. doi:10.1186/s12879-018-3634-3

33. Veland N, Espinosa D, Valencia BM, Ramos AP, Calderon F, Arevalo J, et al. Polymerase chain reaction detection of Leishmania kDNA from the urine of Peruvian patients with cutaneous and mucocutaneous leishmaniasis. The American Journal of Tropical Medicine and Hygiene. 2011;84: 556–561. doi:10.4269/ajtmh.2011.10-0556

34. Adams ER, Jacquet D, Schoone G, Gidwani K, Boelaert M, Cunningham J. Leishmaniasis direct agglutination test: using pictorials as training materials to reduce inter-reader variability and improve accuracy. Büscher P, editor. PLOS Neglected Tropical Diseases. 2012;6: e1946. doi:10.1371/journal.pntd.0001946

35. Mirzaei A. Immunodetection and molecular determination of visceral and cutaneous Leishmania infection using patients’ urine. Infection, Genetics and Evolution. 2018;63: 257–268. doi:10.1016/j.meegid.2018.05.021

36. Zijlstra EE, Sande WWJ van de, Welsh O, Mahgoub ES, Goodfellow M, Fahal AH. Mycetoma: a unique neglected tropical disease. The Lancet Infectious Diseases. 2016;16: 100–112. doi:10.1016/s1473-3099(15)00359-x

37. Emmanuel P, Dumre SP, John S, Karbwang J, Hirayama K. Mycetoma: a clinical dilemma in resource limited settings. Annals of Clinical Microbiology and Antimicrobials. 2018;17. doi:10.1186/s12941-018-0287-4

38. Verma P, Jha A. Mycetoma: reviewing a neglected disease. Clinical and Experimental Dermatology. 2019;44: 123–129. doi:10.1111/ced.13642

39. Ahmed AA, Sande W van de, Fahal AH. Mycetoma laboratory diagnosis: Review article. Clements ACA, editor. PLOS Neglected Tropical Diseases. 2017;11: e0005638. doi:10.1371/journal.pntd.0005638

40. DNDi. Mycetoma: New hope for neglected patients - Developing effective treatments for a truly neglected disease. 2019. Available: https://www.dndi.org/wp-content/uploads/2019/02/DNDi_Mycetoma_2019.pdf

41. Sutherland CS, Stone CM, Steinmann P, Tanner M, Tediosi F. Seeing beyond 2020: an economic evaluation of contemporary and emerging strategies for elimination of Trypanosoma brucei gambiense. The Lancet Global health. 2017;5: e69–e79. doi:10.1016/s2214-109x(16)30237-6

42. WHO. Human African trypanosomiasis (Sleeping sickness). n.d. [cited 2AD]. Available: https://www.who.int/health-topics/human-african-trypanosomiasis#tab=tab_1

43. Bonnet J, Boudot C, Courtioux B. Overview of the Diagnostic Methods Used in the Field for Human African Trypanosomiasis: What Could Change in the Next Years? BioMed Research International. 2015;2015: 583262–10. doi:10.1155/2015/583262

44. Biéler S, Waltenberger H, Barrett MP, McCulloch R, Mottram JC, Carrington M, et al. Evaluation of Antigens for Development of a Serological Test for Human African Trypanosomiasis. Tanowitz HB, editor. PLoS One. 2016;11: e0168074. doi:10.1371/journal.pone.0168074

45. Matovu E, Kazibwe AJ, Mugasa CM, Ndung’u JM, Njiru ZK. Towards Point-of-Care Diagnostic and Staging Tools for Human African Trypanosomiaisis. Journal of tropical medicine. 2012;2012: 340538–9. doi:10.1155/2012/340538

46. Sternberg JM, Gierliński M, Biéler S, Ferguson MAJ, Ndung’u JM. Evaluation of the diagnostic accuracy of prototype rapid tests for human African trypanosomiasis. Raper J, editor. PLOS Neglected Tropical Diseases. 2014;8: e3373. doi:10.1371/journal.pntd.0003373

47. Lumbala C, Biéler S, Kayembe S, Makabuza J, Ongarello S, Ndung’u JM. Prospective evaluation of a rapid diagnostic test for Trypanosoma brucei gambiense infection developed using recombinant antigens. Caljon G, editor. PLOS Neglected Tropical Diseases. 2018;12: e0006386. doi:10.1371/journal.pntd.0006386

48. Boelaert M, Mukendi D, Bottieau E, Lilo JRK, Verdonck K, Minikulu L, et al. A Phase III Diagnostic Accuracy Study of a Rapid Diagnostic Test for Diagnosis of Second-Stage Human African Trypanosomiasis in the Democratic Republic of the Congo. Ebiomedicine. 2018;27: 11–17. doi:10.1016/j.ebiom.2017.10.032

49. Sullivan L, Fleming J, Sastry L, Mehlert A, Wall SJ, Ferguson MAJ. Identification of sVSG117 as an immunodiagnostic antigen and evaluation of a dual-antigen lateral flow test for the diagnosis of human African trypanosomiasis. Raper J, editor. PLOS Neglected Tropical Diseases. 2014;8: e2976. doi:10.1371/journal.pntd.0002976

50. Converse PJ, Xing Y, Kim KH, Tyagi S, Li S-Y, Almeida DV, et al. Accelerated Detection of Mycolactone Production and Response to Antibiotic Treatment in a Mouse Model of Mycobacterium ulcerans Disease. Phillips RO, editor. PLOS Neglected Tropical Diseases. 2014;8: e2618-7. doi:10.1371/journal.pntd.0002618

51. WHO. Management of Buruli Ulcer-Human Immunodeficiency Virus Coinfection. 2015.

52. Sakyi SA, Aboagye SY, Otchere ID, Yeboah-Manu D. Clinical and Laboratory Diagnosis of Buruli Ulcer Disease: A Systematic Review. The Canadian journal of infectious diseases & medical microbiology = Journal canadien des maladies infectieuses et de la microbiologie medicale. 2016;2016: 5310718–10. doi:10.1155/2016/5310718

53. Röltgen K, Cruz I, Ndung’u JM, Pluschke G. Laboratory Diagnosis of Buruli Ulcer: Challenges and Future Perspectives. Springer; 2019. doi:10.1007/978-3-030-11114-4_10

54. WHO, FIND. Report of a WHO–FIND meeting on diagnostics for Buruli ulcer. 2018 Oct pp. 1–19.

55. Diesburg SP, Guelig D, Burton R, Singleton J, Labarre P. Development of a single-use, disposable, electricity-free, nucleic acid amplification platform. GHTC. 2015; 400–406.

56. McManus DP, Dunne DW, Sacko M, Utzinger J, Vennervald BJ, Zhou X-N. Schistosomiasis. Nature reviews Disease primers. 2018;4: 13–19. doi:10.1038/s41572-018-0013-8

57. Nelwan ML. Schistosomiasis: Life Cycle, Diagnosis, and Control. Current therapeutic research, clinical and experimental. 2019;91: 5–9. doi:10.1016/j.curtheres.2019.06.001

58. Hinz R, Schwarz NG, Hahn A, Frickmann H. Serological approaches for the diagnosis of schistosomiasis - A review. Molecular and cellular probes. 2017;31: 2–21. doi:10.1016/j.mcp.2016.12.003

59. Hotez PJ, Alvarado M, Basáñez M-G, Bolliger I, Bourne R, Boussinesq M, et al. The Global Burden of Disease Study 2010: Interpretation and Implications for the Neglected Tropical Diseases. Silva N de, editor. PLOS Neglected Tropical Diseases. 2014;8: e2865. doi:10.1371/journal.pntd.0002865

60. Utzinger J, Becker SL, Lieshout LV, Dam GJV, Knopp S. New diagnostic tools in schistosomiasis. Clinical Microbiology and Infection. 2015;21: 529–542. doi:10.1016/j.cmi.2015.03.014

61. Cools P, Vlaminck J, Albonico M, Ame S, Ayana M, Antonio BPJ, et al. Diagnostic performance of a single and duplicate Kato-Katz, Mini-FLOTAC, FECPAKG2 and qPCR for the detection and quantification of soil-transmitted helminths in three endemic countries. Freeman MC, editor. PLOS Neglected Tropical Diseases. 2019;13: e0007446. doi:10.1371/journal.pntd.0007446

62. Verweij JJ, Stensvold CR. Molecular Testing for Clinical Diagnosis and Epidemiological Investigations of Intestinal Parasitic Infections. Clinical microbiology reviews. 2014;27: 371–418. doi:10.1128/cmr.00122-13

63. Legesse M, Erko B. Field-based evaluation of a reagent strip test for diagnosis of Schistosoma mansoni by detecting circulating cathodic antigen in urine before and after chemotherapy. Transactions of the Royal Society of Tropical Medicine and Hygiene. 2007;101: 668–673. doi:10.1016/j.trstmh.2006.11.009

64. Colley DG, King CH, Kittur N, Ramzy RMR, Secor WE, Fredericks-James M, et al. Evaluation, Validation, and Recognition of the Point-of-Care Circulating Cathodic Antigen, Urine-Based Assay for Mapping Schistosoma mansoni Infections. Am J Tropical Medicine Hyg. 2020;103: 42–49. doi:10.4269/ajtmh.19-0788

65. Beltrame A, Guerriero M, Angheben A, Gobbi F, Requena-Mendez A, Zammarchi L, et al. Accuracy of parasitological and immunological tests for the screening of human schistosomiasis in immigrants and refugees from African countries: An approach with Latent Class Analysis. Plos Neglect Trop D. 2017;11: e0005593. doi:10.1371/journal.pntd.0005593

66. Agbana T, Van GY, Oladepo O, Vdovin G, Oyibo W, Diehl JC. Schistoscope - Towards a locally producible smart diagnostic device for Schistosomiasis in Nigeria. GHTC. 2019; 1–8. doi:10.1109/ghtc46095.2019.9033049

67. Rassi A, Marin-Neto JA. Chagas disease. Lancet. 2010;375: 1388–1402. doi:10.1016/s0140-6736(10)60061-x

68. Coura JR, Viñas PA. Chagas disease: a new worldwide challenge. Nature. 2010;465: S6–S7. doi:10.1038/nature09221

69. Collaborators G 2016 Daly and H. Global, Regional, and National Disability-adjusted Life-years (DALYs) for 333 Diseases and Injuries and Healthy Life Expectancy (HALE) for 195 Countries and Territories, 1990–2016: a Systematic Analysis for the Global Burden of Disease Study 2016. 2017. doi:10.1016/s0140-6736(17)32130-x

70. Schijman AG, Vigliano C, Burgos J, Favaloro R, Perrone S, Laguens R, et al. Early diagnosis of recurrence of Trypanosoma cruzi infection by polymerase chain reaction after heart transplantation of a chronic Chagas’ heart disease patient. The Journal of heart and lung transplantation : the official publication of the International Society for Heart Transplantation. 2000;19: 1114–1117. doi:10.1016/s1053-2498(00)00168-6

71. Sosa-Estani S, Viotti R, Segura EL. Therapy, diagnosis and prognosis of chronic Chagas disease: insight gained in Argentina. Memorias do Instituto Oswaldo Cruz. 2009;104 Suppl 1: 167–180. doi:10.1590/s0074-02762009000900023

72. Sánchez-Camargo CL, Albajar-Viñas P, Wilkins PP, Nieto J, Leiby DA, Paris L, et al. Comparative evaluation of 11 commercialized rapid diagnostic tests for detecting Trypanosoma cruzi antibodies in serum banks in areas of endemicity and nonendemicity. Gilligan PH, editor. Journal of Clinical Microbiology. 2014;52: 2506–2512. doi:10.1128/jcm.00144-14

73. Ponce C, Ponce E, Vinelli E, Montoya A, Aguilar V de, Gonzalez A, et al. Validation of a Rapid and Reliable Test for Diagnosis of Chagas’ Disease by Detection of Trypanosoma cruzi-Specific Antibodies in Blood of Donors and Patients in Central America. Journal of Clinical Microbiology. 2005;43: 5065–5068. doi:10.1128/jcm.43.10.5065-5068.2005

74. Shah V, Ferrufino L, Gilman RH, Ramirez M, Saenza E, Malaga E, et al. Field Evaluation of the InBios Chagas Detect Plus Rapid Test in Serum and Whole-Blood Specimens in Bolivia. Wilkins PP, editor. Clinical and vaccine immunology : CVI. 2014;21: 1645–1649. doi:10.1128/cvi.00609-14

75. Balouz V, Agüero F, Buscaglia CA. Chapter One Chagas Disease Diagnostic Applications Present Knowledge and Future Steps. Adv Parasit. 2017;97: 1–45. doi:10.1016/bs.apar.2016.10.001

76. Inc. GI, Berger DS. Visceral Leishmaniasis: Global Status. GIDEON Informatics Inc; 2019.

77. Organization WH. Control of the leishmaniases. World Health Organization. 2010.

78. CDC. Parasites - Leishmaniasis. n.d.

79. Tlamcani Z. Visceral leishmaniasis: an update of laboratory diagnosis. Asian Pacific Journal of Tropical Disease. 2016;6: 505–508. doi:10.1016/s2222-1808(16)61077-0

80. Sakkas H, Gartzonika C, Levidiotou S. Laboratory diagnosis of human visceral leishmaniasis. Journal of Vector Borne Diseases. 2016;53: 8–16.

81. Akhoundi M, Downing T, Votýpka J, Kuhls K, Lukeš J, Cannet A, et al. Leishmania infections: Molecular targets and diagnosis. Molecular Aspects of Medicine. 2017;57: 1–29. doi:10.1016/j.mam.2016.11.012

82. Adams ER, Schoone G, Versteeg I, Gomez MA, Diro E, Mori Y, et al. Development and Evaluation of a Novel Loop-Mediated Isothermal Amplification Assay for Diagnosis of Cutaneous and Visceral Leishmaniasis. Loeffelholz MJ, editor. Journal of Clinical Microbiology. 2018;56: S102. doi:10.1128/jcm.00386-18

83. Boelaert M, El-Safi S, Hailu A, Mukhtar M, Rijal S, Sundar S, et al. Diagnostic tests for kala-azar: a multi-centre study of the freeze-dried DAT, rK39 strip test and KAtex in East Africa and the Indian subcontinent. Transactions of the Royal Society of Tropical Medicine and Hygiene. 2008;102: 32–40. doi:10.1016/j.trstmh.2007.09.003

84. Singh OP, Sundar S. Developments in diagnosis of visceral leishmaniasis in the elimination era. Journal of Parasitology Research. 2015;2015: 239469. doi:10.1155/2015/239469

85. Vaish M, Mehrotra S, Chakravarty J, Sundar S. Noninvasive molecular diagnosis of human visceral leishmaniasis. Journal of Clinical Microbiology. 2011;49: 2003–2005. doi:10.1128/jcm.00130-11

86. Vaish M, Singh OP, Chakravarty J, Sundar S. rK39 antigen for the diagnosis of visceral leishmaniasis by using human saliva. The American Journal of Tropical Medicine and Hygiene. 2012;86: 598–600. doi:10.4269/ajtmh.2012.11-0127

87. Bezuneh A, Mukhtar M, Abdoun A, Teferi T, Takele Y, Diro E, et al. Comparison of point-of-care tests for the rapid diagnosis of visceral leishmaniasis in East African patients. The American Journal of Tropical Medicine and Hygiene. 2014;91: 1109–1115. doi:10.4269/ajtmh.13-0759

88. Humbert MV, Costa LE, Katis I, Ramos FF, Machado AS, Sones C, et al. A rapid diagnostic test for human Visceral Leishmaniasis using novel Leishmania antigens in a Laser Direct-Write Lateral Flow Device. Emerging microbes & infections. 2019;8: 1178–1185. doi:10.1080/22221751.2019.1635430

89. Matlashewski G, Das VNR, Pandey K, Singh D, Das S, Ghosh AK, et al. Diagnosis of visceral leishmaniasis in Bihar India: comparison of the rK39 rapid diagnostic test on whole blood versus serum. Debrabant A, editor. PLOS Neglected Tropical Diseases. 2013;7: e2233. doi:10.1371/journal.pntd.0002233

90. Mondal D, Ghosh P, Khan MAA, Hossain F, Böhlken-Fascher S, Matlashewski G, et al. Mobile suitcase laboratory for rapid detection of Leishmania donovani using recombinase polymerase amplification assay. Parasites & Vectors. 2016;9: 281–8. doi:10.1186/s13071-016-1572-8

91. Koltas IS, Eroglu F, Uzun S, Alabaz D. A comparative analysis of different molecular targets using PCR for diagnosis of old world leishmaniasis. Experimental Parasitology. 2016;164: 43–48. doi:10.1016/j.exppara.2016.02.007

92. Taylor MJ, Hoerauf A, Bockarie M. Lymphatic filariasis and onchocerciasis. Lancet. 2010;376: 1175–1185. doi:10.1016/s0140-6736(10)60586-7

93. cdc. Parasites - Lymphatic Filariasis. n.d.

94. WHO. Lymphatic filariasis : fourth report of the WHO Expert Committee on Filariasis [‎meeting held in Geneva from 31 October to 8 November 1983]. 1984 p. 112. Available: https://apps.who.int/iris/handle/10665/39063

95. Rebollo MP, Bockarie MJ. Can Lymphatic Filariasis Be Eliminated by 2020? Trends in Parasitology. 2017;33: 83–92. doi:10.1016/j.pt.2016.09.009

96. Hertz MI, Nana-Djeunga H, Kamgno J, Njouendou AJ, Chunda VC, Wanji S, et al. Identification and characterization of Loa loa antigens responsible for cross-reactivity with rapid diagnostic tests for lymphatic filariasis. Specht S, editor. PLOS Neglected Tropical Diseases. 2018;12: e0006963. doi:10.1371/journal.pntd.0006963

97. Natrajan MS, Rojas A, Waggoner JJ. Beyond Fever and Pain: Diagnostic Methods for Chikungunya Virus. Kraft CS, editor. Journal of Clinical Microbiology. 2019;57: 209. doi:10.1128/jcm.00350-19

98. Bloch D. The Cost And Burden Of Chikungunya In The Americas. 1AD.

99. CDC. Chikungunya Virus. n.d. [cited 5 Apr 2021]. Available: https://www.cdc.gov/chikungunya/index.html

100. Hayashida K, Orba Y, Sequeira PC, Sugimoto C, Hall WW, Eshita Y, et al. Field diagnosis and genotyping of chikungunya virus using a dried reverse transcription loop-mediated isothermal amplification (LAMP) assay and MinION sequencing. Kittayapong P, editor. PLOS Neglected Tropical Diseases. 2019;13: e0007480. doi:10.1371/journal.pntd.0007480

101. Vongsouvath M, Bharucha T, Seephonelee M, Lamballerie X de, Newton PN, Dubot-Pérès A. Harnessing Dengue Rapid Diagnostic Tests for the Combined Surveillance of Dengue, Zika, and Chikungunya Viruses in Laos. The American Journal of Tropical Medicine and Hygiene. 2020;102: 1244–1248. doi:10.4269/ajtmh.19-0881

102. Johnston G, Sladden M. Scabies: diagnosis and treatment. BMJ (Clinical research ed). 2005;331: 619–622. doi:10.1136/bmj.331.7517.619

103. Kinyanjui T, Middleton J, Güttel S, Cassell J, Ross J, House T. Scabies in residential care homes: Modelling, inference and interventions for well-connected population sub-units. Alizon S, editor. PLoS computational biology. 2018;14: e1006046. doi:10.1371/journal.pcbi.1006046

104. Leung V, Miller M. Detection of Scabies: A Systematic Review of Diagnostic Methods. Canadian Journal of Infectious Diseases and Medical Microbiology. 2011;22: 143–146. doi:10.1155/2011/698494

105. Engelman D, Fuller LC, Steer AC, Scabies IAC. Consensus criteria for the diagnosis of scabies: A Delphi study of international experts. Vinetz JM, editor. PLOS Neglected Tropical Diseases. 2018;12. doi:10.1371/journal.pntd.0006549

106. WHO. Ending the neglect to attain the sustainable development goals: a road map for neglected tropical diseases 2021–2030. Geneva: World Health Organization. 2020. Licence:  CC BY-NC-SA 3.0 IGO.

107. Park JH, Kim CW, Kim SS. The diagnostic accuracy of dermoscopy for scabies. Annals of dermatology. 2012;24: 194–199. doi:10.5021/ad.2012.24.2.194

108. Colebunders R, Stolk WA, Fodjo JNS, Mackenzie CD, Hopkins A. Elimination of onchocerciasis in Africa by 2025: an ambitious target requires ambitious interventions. Infectious Diseases of Poverty. 2019;8: 83. doi:10.1186/s40249-019-0593-x

109. Puri PK. Onchocerciasis. Dermatopathology Diagnosis. 2015; 1–3. Available: https://mdedge-files-live.s3.us-east-2.amazonaws.com/files/s3fs-public/issues/articles/CT095030131.pdf

110. Inc. GI, Berger DS. Onchocerciasis: Global Status. GIDEON Informatics Inc; 2020.

111. Hopkins A. Onchocerciasis: the beginning of the end? Community eye health. 2019;31: 94.

112. Unnasch TR, Golden A, Cama V, Cantey PT. Diagnostics for onchocerciasis in the era of elimination. International Health. 2018;10: i20–i26. doi:10.1093/inthealth/ihx047

113. Steel C, Golden A, Stevens E, Yokobe L, Domingo GJ, Santos T de los, et al. Rapid Point-of-Contact Tool for Mapping and Integrated Surveillance of Wuchereria bancrofti and Onchocerca volvulus Infection. Wilkins PP, editor. Clinical and vaccine immunology : CVI. 2015;22: 896–901. doi:10.1128/cvi.00227-15

114. Chappuis F, Loutan L, Simarro P, Lejon V, Büscher P. Options for Field Diagnosis of Human African Trypanosomiasis. Clin Microbiol Rev. 2005;18: 133–146. doi:10.1128/cmr.18.1.133-146.2005

115. Benjamin JM, Abo BN, Brandehoff N. Review Article: Snake Envenomation in Africa. Current Tropical Medicine Reports. 2020;7: 1–10. doi:10.1007/s40475-020-00198-y

116. Williams HF, Layfield HJ, Vallance T, Patel K, Bicknell AB, Trim SA, et al. The Urgent Need to Develop Novel Strategies for the Diagnosis and Treatment of Snakebites. Toxins. 2019;11: 363. doi:10.3390/toxins11060363

117. Laxme RRS, Khochare S, Souza HF de, Ahuja B, Suranse V, Martin G, et al. Beyond the “big four”: Venom profiling of the medically important yet neglected Indian snakes reveals disturbing antivenom deficiencies. BILLIALD P, editor. PLOS Neglected Tropical Diseases. 2019;13: e0007899. doi:10.1371/journal.pntd.0007899

118. O’Rourke KM, Correlje E, Martin CL, Robertson JD, Isbister GK. Point-of-care derived INR does not reliably detect significant coagulopathy following Australian snakebite. Thrombosis research. 2013;132: 610–613. doi:10.1016/j.thromres.2013.09.004

119. Wilder-Smith A, Ooi E-E, Horstick O, Wills B. Dengue. Lancet. 2019;393: 350–363.

120. Beatty ME, Beutels P, Meltzer MI, Shepard DS, Hombach J, Hutubessy R, et al. Health Economics of Dengue: A Systematic Literature Review and Expert Panel’s Assessment. Am J Tropical Medicine Hyg. 2011;84: 473–488. doi:10.4269/ajtmh.2011.10-0521

121. CDC. Dengue. n.d. [cited 1AD]. Available: https://www.cdc.gov/dengue/index.html

122. Gan VC, Tan L-K, Lye DC, Pok K-Y, Mok S-Q, Chua RC-R, et al. Diagnosing dengue at the point-of-care: utility of a rapid combined diagnostic kit in Singapore. Bausch DG, editor. PLoS One. 2014;9: e90037. doi:10.1371/journal.pone.0090037

123. Wang SM, Sekaran SD. Early diagnosis of Dengue infection using a commercial Dengue Duo rapid test kit for the detection of NS1, IGM, and IGG. The American Journal of Tropical Medicine and Hygiene. 2010;83: 690–695. doi:10.4269/ajtmh.2010.10-0117

124. Blacksell SD, Jarman RG, Bailey MS, Tanganuchitcharnchai A, Jenjaroen K, Gibbons RV, et al. Evaluation of six commercial point-of-care tests for diagnosis of acute dengue infections: the need for combining NS1 antigen and IgM/IgG antibody detection to achieve acceptable levels of accuracy. Clinical and vaccine immunology : CVI. 2011;18: 2095–2101. doi:10.1128/cvi.05285-11

125. Buyon L, Slaven R, Emerson PM, King J, Debrah O, Aboe A, et al. Achieving the endgame: Integrated NTD case searches. Ngondi JM, editor. PLOS Neglected Tropical Diseases. 2018;12: e0006623. doi:10.1371/journal.pntd.0006623

126. Cairncross S, Muller R, Zagaria N. Dracunculiasis (guinea worm disease) and the eradication initiative. Clinical microbiology reviews. 2002;15: 223-+. doi:10.1128/cmr.15.2.223-246.2002

127. Boyce MR, Carlin EP, Schermerhorn J, Standley CJ. A One Health Approach for Guinea Worm Disease Control: Scope and Opportunities. Tropical Medicine Infect Dis. 2020;5: 159. doi:10.3390/tropicalmed5040159

128. Belachew WA, Naafs B. Position statement: Leprosy: Diagnosis, treatment and follow-up. Journal of the European Academy of Dermatology and Venereology : JEADV. 2019;33: 1205–1213. doi:10.1111/jdv.15569

129. Fischer M. Leprosy – an overview of clinical features, diagnosis, and treatment. JDDG: Journal der Deutschen Dermatologischen Gesellschaft. 2017;15: 801–827. doi:10.1111/ddg.13301

130. Mitra AK, Mawson AR. Neglected Tropical Diseases: Epidemiology and Global Burden. Tropical medicine and infectious disease. 2017;2: 36. doi:10.3390/tropicalmed2030036

131. Reibel F, Cambau E, Aubry A. Update on the epidemiology, diagnosis, and treatment of leprosy. Médecine et Maladies Infectieuses. 2015;45: 383–393. doi:10.1016/j.medmal.2015.09.002

132. Hooij A van, Eeden S van den, Richardus R, Fat ETK, Wilson L, Franken KLMC, et al. Application of new host biomarker profiles in quantitative point-of-care tests facilitates leprosy diagnosis in the field. Ebiomedicine. 2019;47: 301–308. doi:10.1016/j.ebiom.2019.08.009

133. Ngwese MM, Manouana GP, Moure PAN, Ramharter M, Esen M, Adégnika AA. Diagnostic Techniques of Soil-Transmitted Helminths: Impact on Control Measures. Tropical Medicine Infect Dis. 2020;5: 93. doi:10.3390/tropicalmed5020093

134. Yang A, Bakhtari N, Langdon-Embry L, Redwood E, Lapierre SG, Rakotomanga P, et al. Kankanet: An artificial neural network-based object detection smartphone application and mobile microscope as a point-of-care diagnostic aid for soil-transmitted helminthiases. Bennuru S, editor. PLOS Neglected Tropical Diseases. 2019;13: e0007577. doi:10.1371/journal.pntd.0007577

135. Mutombo PN, Man NWY, Nejsum P, Ricketson R, Gordon CA, Robertson G, et al. Diagnosis and drug resistance of human soil-transmitted helminth infections: A public health perspective. Adv Parasit. 2019;104: 247–326. doi:10.1016/bs.apar.2019.02.004

136. Solomon AW, Holland MJ, Alexander NDE, Massae PA, Aguirre A, Natividad-Sancho A, et al. Mass treatment with single-dose azithromycin for trachoma. The New England journal of medicine. 2004;351: 1962–1971. doi:10.1056/nejmoa040979

137. Mohammadpour M, Abrishami M, Masoumi A, Hashemi H. Trachoma: Past, present and future. Journal of current ophthalmology. 2016;28: 165–169. doi:10.1016/j.joco.2016.08.011

138. Gwyn S, Mitchell A, Dean D, Mkocha H, Handali S, Martin DL. Lateral flow-based antibody testing for Chlamydia trachomatis. Journal of immunological methods. 2016;435: 27–31. doi:10.1016/j.jim.2016.05.008

139. Solomon AW, Mesurier RTL, Williams WJ. A diagnostic instrument to help field graders evaluate active trachoma. Ophthalmic epidemiology. 2018;25: 399–402. doi:10.1080/09286586.2018.1500616

140. Harding-Esch EM, Holland MJ, Schémann J-F, Molina S, Sarr I, Andreasen AA, et al. Diagnostic accuracy of a prototype point-of-care test for ocular Chlamydia trachomatis under field conditions in The Gambia and Senegal. Schachter J, editor. PLOS Neglected Tropical Diseases. 2011;5: e1234. doi:10.1371/journal.pntd.0001234

141. Mitjà O, Asiedu K, Mabey D. Yaws. Lancet. 2013;381: 763–773. doi:10.1016/s0140-6736(12)62130-8

142. Giacani L, Lukehart SA. The Endemic Treponematoses. Clinical microbiology reviews. 2014;27: 89–115. doi:10.1128/cmr.00070-13

143. Fitzpatrick C, Asiedu K, Jannin J. Where the road ends, yaws begins? The cost-effectiveness of eradication versus more roads. Lukehart S, editor. PLOS Neglected Tropical Diseases. 2014;8: e3165. doi:10.1371/journal.pntd.0003165

144. Marks M, Goncalves A, Vahi V, Sokana O, Puiahi E, Zhang Z, et al. Evaluation of a rapid diagnostic test for yaws infection in a community surveillance setting. Phillips RO, editor. PLOS Neglected Tropical Diseases. 2014;8: e3156. doi:10.1371/journal.pntd.0003156

145. Chi K-H, Danavall D, Taleo F, Pillay A, Ye T, Nachamkin E, et al. Molecular Differentiation of Treponema pallidum Subspecies in Skin Ulceration Clinically Suspected as Yaws in Vanuatu Using Real-Time Multiplex PCR and Serological Methods. The American Journal of Tropical Medicine and Hygiene. 2015;92: 134–138. doi:10.4269/ajtmh.14-0459

146. Yin Y-P, Chen X-S, Wei W-H, Gong K-L, Cao W-L, Yong G, et al. A Dual Point-of-Care Test Shows Good Performance in Simultaneously Detecting Nontreponemal and Treponemal Antibodies in Patients With Syphilis: A Multisite Evaluation Study in China. Clinical infectious diseases : an official publication of the Infectious Diseases Society of America. 2013;56: 659–665. doi:10.1093/cid/cis928

147. Basing LAW, Simpson SV, Adu-Sarkodie Y, Linnes JC. A Loop-Mediated Isothermal Amplification Assay for the Detection of Treponema pallidum subsp. pertenue. The American Journal of Tropical Medicine and Hygiene. 2020; tpmd190243. doi:10.4269/ajtmh.19-0243

1. Note that ‘antigen-based tests’ use antigens in the test to detect antibodies in a sample. This contrasts to ‘antibody-based tests’ that detect antigens in a sample. [↑](#footnote-ref-1)
